# Techno-Economic Analysis of Biogas-to-Methanol via Integrated Oxy-Combustion and Electrolytic Routes

**DOI:** 10.1021/acs.iecr.6c00107

**Published:** 2026-06-24

**Authors:** Mohammad Jafari-Mohsen-Abad, Massimiliano Toto, Federico d’Amore, Elena Barbera, Fabrizio Bezzo

**Affiliations:** 1 CAPE-Lab − Computer-Aided Process Engineering Laboratory, Department of Industrial Engineering, 165501 University of Padova, via Marzolo 9, Padova, PD, 35131, Italy; ‡ INSTM − National Interuniversity Consortium of Materials Science and Technology, Via G. Giusti 9, Firenze, FI 44121, Italy; 3 Incico S.p.A., via Terranuova, 28, Ferrara (FE), 44121, Italy; 4 BiERLab − Bioprocess Engineering Laboratory, Department of Industrial Engineering, 165501 University of Padova, Via Marzolo 9, Padova, PD, 35131, Italy

## Abstract

This study presents a techno-economic analysis of two biogas-to-methanol process designs via integrated oxy-fuel combined heat and power and either Solid Oxide Electrolysis Cells (SOEC) or Alkaline Water Electrolysis (AWE). Both routes are modeled for an inlet biogas flow of 2000 Nm^3^/h (60%_mol_ CH_4_, 40%_mol_ CO_2_ composition). Results reveal that the AWE route achieves a superior carbon conversion efficiency (88.8%), resulting in a higher annual production of chemical-grade methanol (19.7 kt/year) compared to the SOEC counterpart, this exhibiting a carbon efficiency of 76.6% and an annual methanol production of 17.5 kt/year. Oppositely, the SOEC route exhibits lower specific electric consumptions per unit of methanol produced (6.49 MWh_el_/t of methanol, against 7.45 MWh_el_/t of methanol for the AWE route). Under assumptions compatible with the Italian context, the SOEC route has better economic performance, achieving a net present value of −105.2 M€, compared to −133.4 M€ for the AWE case. Breakeven methanol prices are found equal to 1919 €/t for SOEC and 2020 €/t for AWE, significantly above the reference fossil-based methanol (884 €/t). A spatial analysis across 27 European countries identifies the electricity price and the grid carbon intensity as the dominant cost drivers, as the economic competitiveness of such technologies is shown to require a combination of low-cost renewable electricity and premium methanol pricing to achieve net positive returns.

## Graphical abstract

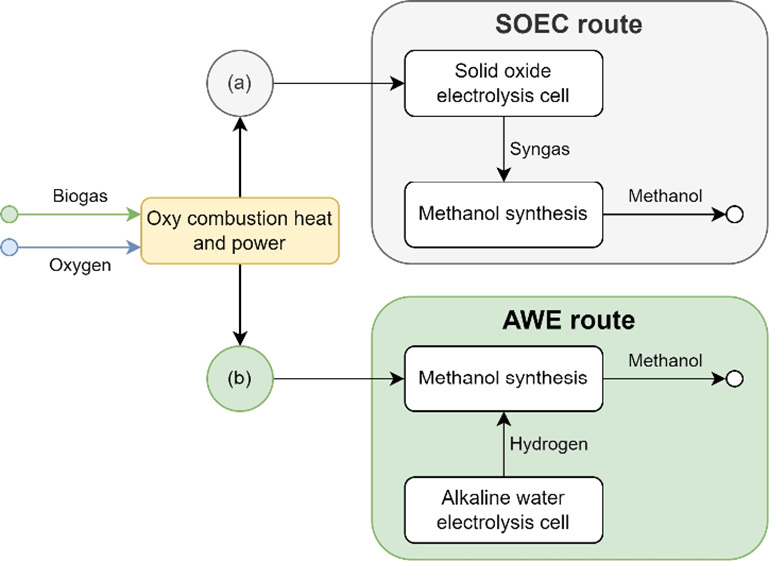

## 1 Introduction

The need to mitigate climate change requires a change in industrial production processes toward low-carbon systems,^1^ particularly for the chemical industry, characterized by substantial levels of CO_2_ emissions on a global scale.^2^ In this context, methanol is an important bulk chemical that accounts for a considerable quota of greenhouse gas emissions.^3^ It is the fundamental building block for a wide range of products, including formaldehyde and acetic acid, and its demand is expected to increase following its potential use as an alternative energy carrier. This phenomenon is represented by the global demand for methanol, which surpassed 100 million metric tons (t) in 2021 and is projected to continue its upward trend.^4^ Conventional methanol synthesis follows a three-step process: syngas generation, methanol synthesis, and product purification. In particular, steam methane reforming of natural gas is currently the most deployed technology,^5,6^ and it represents an inherently carbon-intensive step due to the generation of both process emissions (from the reforming and water–gas shift of methane) and energy-related ones (from the combustion of methane and process off-gases).^7–10^ As such, research is addressing the design of alternative, low-carbon methanol production routes, also in view of its potential market expansion as an alternative fuel,^11^ e.g., in the maritime sector.^12,13^

Sustainable pathways for low-carbon methanol production can be broadly categorized into two main groups, namely biomethanol and electro-methanol routes. In the former, methanol derives from biogenic feedstock typically through biogas reforming or biomass gasification.^14,15^ Recent studies investigating biogas-to-methanol production based on anaerobic digestion and downstream chemical conversion have demonstrated the technical feasibility of green methanol production without electrolysis integration,^16^ providing a useful benchmark for electrolysis-enhanced configurations. As an alternative, electro-methanol routes rely on carbon dioxide (CO_2_) hydrogenation via hydrogen (H_2_) from electrolytic processes,^11,17^ such as low-temperature alkaline water electrolysis (AWE) or high-temperature solid oxide electrolysis cells (SOEC).^18^ This work integrates a biogenic feed of carbon from biogas with electrolytic processes for methanol production, to compare two distinct technological options, SOEC and AWE. SOECs operate at high temperatures, enabling direct coelectrolysis of steam and CO_2_ into syngas with controllable H_2_/CO ratios and the potential to enhance the overall energy efficiency through heat recovery.^19,20^ Comprehensive reviews on SOEC process integration emphasize its potential for industrial symbiosis and high-efficiency coupling with synthetic fuel production processes.^21^ Conversely, AWE is a more mature, low-temperature technology to produce H_2_ and oxygen (O_2_) streams from water (H_2_O). The two technologies involve trade-offs in efficiency, flexibility, and costs,^22^ justifying a detailed comparative assessment of their corresponding routes for sustainable methanol synthesis.

The increasing interest in low-carbon methanol has stimulated a growing body of research on both biomethanol and electro-methanol process designs,^23,24^ with several studies focusing on individual process units^25^ and others on integrated plant configurations.^8,26^ Early contributions examined the feasibility of producing methanol via CO_2_ hydrogenation using electrolytic hydrogen. Van-Dal and Bouallou^7^ demonstrated through process simulation that electro-methanol synthesis can achieve significant CO_2_ avoidance when the oxygen byproduct is valorized, for instance, in combustion-related applications. Their work highlighted the strong dependence of economic performance on electricity prices and underscored the need for a low-carbon electricity supply, since process emissions are largely driven by electricity consumption.^27,28^ This established the basis for later studies that explored renewable CO_2_ sources and more integrated process designs. In parallel, efforts have been made to address the challenge of supplying CO_2_ of adequate purity and the environmental benefits of exploiting carbon from biogenic sources to maximize the overall CO_2_ avoidance performance of the final methanol product.^23,24^ Schorn et al.^14^ investigated an oxy-fuel combustion concept applied to biogas engines within a Power-to-X context, showing that decentralized biogenic CO_2_ can be produced at competitive separation costs. Their findings demonstrate that biogas plants may serve as a reliable biogenic CO_2_ source for methanol synthesis and motivated the exploration of integrated biomethanol and electro-methanol routes (i.e., Power and Biomass-to-X).^29^ Recent work has explored the integration of biogas conversion with solid oxide electrolysis for bio-e-methanol production, demonstrating improved energy efficiency through partial oxidation and tailored heat integration strategies.^30^ Integrated configurations combining biomass-to-methanol processes with SOEC and electric power generation have also been assessed, highlighting the influence of electricity coproduction on overall plant performance.^31^ For instance, the integration of anaerobic digestion with SOEC and carbon-capture-and-utilization (CCU) frameworks, including oxy-fuel power generation, has recently been assessed from techno-economic, exergy, and life-cycle perspectives, confirming the potential of electrolysis-enhanced biofuel systems,^32^ as well as coutilization strategies, combining biogenic carbon and captured CO_2_ streams, have highlighted potential for improving cost and sustainability performances in methanol production.^33^ Renewable methanol production from waste-derived carbon integrated with oxy-fuel combustion and electrolysis has also been evaluated through combined techno-economic and life-cycle assessment frameworks.^34^ Parallelly, approaches combining biogas reforming with electrolytic hydrogen have shown improved carbon utilization while revealing trade-offs in design and costs.^35^

Research has also addressed the comparative assessment of electrolysis technologies and syngas generation pathways, particularly for SOEC systems.^36,37^ Mbatha et al.^38^ and Gupta et al.^20^ showed that SOEC-based processes can reach high energy efficiencies due to the thermodynamic benefits of high-temperature operation, in agreement with system-level modeling results for Power-to-Methanol.^26,39^ These findings reinforce the potential of high-temperature electrolysis, particularly when syngas ratios can be tailored through coelectrolysis for downstream methanol synthesis. Alongside technical analyses, several economic and life-cycle studies have underscored the decisive role of electricity use in both operating costs and the carbon footprint of low-carbon methanol routes.^8,24,27,28^ Rajaee et al.^40^ evaluated biomass-to-methanol concepts incorporating SOEC technologies and reported that configurations based on direct biosyngas electrolysis achieve the lowest production costs, whereas CO_2_–H_2_O coelectrolysis options are penalized by higher energy consumption and capital investment. Parallelly, Scomazzon et al.^15^ showed that utilities, especially electricity for electrolysis and CO_2_ capture, account for a large share of operating expenses in CO_2_ hydrogenation plants, with renewable methanol production costs remaining significantly above those of fossil-based methanol under current price assumptions. These studies collectively underline that the competitiveness of emerging biogas-to-methanol routes hinges on both process efficiency and access to low-cost renewable electricity.

However, while previous studies have investigated SOEC-based power-to-methanol optimization,^18^ biogas-to-methanol routes based on anaerobic digestion,^16^ hybrid reforming configurations,^35^ and various integrations of biogenic carbon sources with electrolysis and methanol synthesis, a consistent side-by-side techno-economic comparison of high-temperature SOEC coelectrolysis and low-temperature AWE-based CO_2_ hydrogenation within an integrated biogas-to-methanol framework remains lacking. The present work proposes the techno-economic analysis of a biogas-to-methanol process configuration that integrates oxy-fuel combustion with high-temperature SOEC (i.e., SOEC route), coupled with the oxy-fuel combustion of biogas for the combined generation of heat and electricity (CHP) and simultaneous production of a high-purity CO_2_ stream, which is useful for methanol synthesis.^14^ The high-temperature electrolysis could potentially result in a lower energy demand per unit of output methanol produced. This study evaluates the techno-economic and environmental performance of the proposed SOEC route in comparison with an AWE one, aiming to understand the potential of integrated CHP electrolysis systems for low-carbon methanol production. The overreaching objective is to undertake a critical comparison of the SOEC and AWE routes within the biogas-to-methanol context. Accordingly, the research questions addressed in this study are (*i*) which process configuration between SOEC and AWE routes may offer superior techno-economic performance? (*ii*) How do the environmental impacts in terms of CO_2_ avoidance of SOEC and AWE routes compare under consistent assumptions? (*iii*) What process parameters influence the most the economic viability of the proposed processes? The objectives of this study are (*i*) to develop and model two alternative biogas-to-methanol process configurations, integrating SOEC coelectrolysis or AWE-based H_2_ generation, together with oxy-fuel combustion; (*ii*) to evaluate and compare the techno-economic performance, energy efficiency, and environmental impacts of the two configurations under consistent assumptions; (*iii*) to identify the key process parameters affecting efficiency, cost, and CO_2_ emissions.

The article is organized as follows. Section 2 introduces the overall process concepts through simplified block diagrams, followed by a detailed description of the full plant schemes. Section 3 outlines the methodology, key assumptions, modeling parameters, and performance metrics. Section 4 presents the results in terms of techno-economic performance and geographic sensitivity across Europe. Section 5 summarizes the key findings and concludes the work.

## 2 Process Description

In this study, two biogas-to-methanol routes are modeled and simulated in Aspen+ V.14 software. Both configurations are designed to produce chemical-grade methanol (>99.5%_wt_) from an inlet biogas flow rate of 2000 Nm^3^/h, with a molar composition of 60%_mol_ CH_4_ and 40%_mol_ of CO_2_, which represents the output of a medium-size biogas plant.^41^ The inlet biogas stream is assumed to be pretreated through conventional upgrading processes (e.g., H_2_S and NH_3_ removal) prior to entering the plant, consistently with industrial practice, and the system boundary of this study is defined downstream of gas conditioning. The simplified block-flow diagrams of the SOEC- and AWE-based routes are shown in Figure 1. Both processes comprise an oxy-fuel combined heat and power (CHP) plant, where combustion of biogas with pure oxygen from electrolysis takes place, while they differ in terms of electrolysis technology:

- High-temperature SOEC route (Figure 1a). In this configuration, the hot flue gas from oxy-combustion, comprising CO_2_ and steam, is partly recycled to the boiler with additional steam (to moderate combustion temperature), while the remainder is fed to the SOEC unit, where it undergoes coelectrolysis at 800 °C and atmospheric pressure to produce syngas (i.e., CO and H_2_). The syngas is subsequently conditioned by drying and compression to reach reaction pressure (75 bar) for methanol production. Products of methanol synthesis go through a purification section, consisting of a high-pressure flash separator and a multistage distillation train, where high-purity methanol (>99.5%_wt_) is produced. This process configuration is characterized by a significant degree of thermal integration, which is achieved by keeping the inlet stream of SOEC at the same operating temperature of the cell, and by extracting the remaining available heat to produce electricity via a Rankine cycle in the CHP plant.
- Low-temperature AWE route (Figure 1b). Also in this case, the hot flue gas products of oxy-combustion are exploited to generate electricity. The resulting flue gas is cooled and dried to obtain high-purity CO_2_, which is partly recycled to the combustion chamber to moderate the temperature, while the remainder is sent to the methanol synthesis section. Parallelly, an AWE unit operates at a temperature of 70 °C and a pressure of 10 bar to produce H_2_ and O_2_ streams. The oxygen is sent to the CHP plant for combustion, while the hydrogen is exploited for methanol synthesis alongside CO_2_. The products from this reaction subsequently go through a separation and purification section, consisting of flash separation and distillation columns, where high-purity methanol (99.5%_wt_) is achieved, consistently with the target purity of the SOEC route.

**Figure 1:**
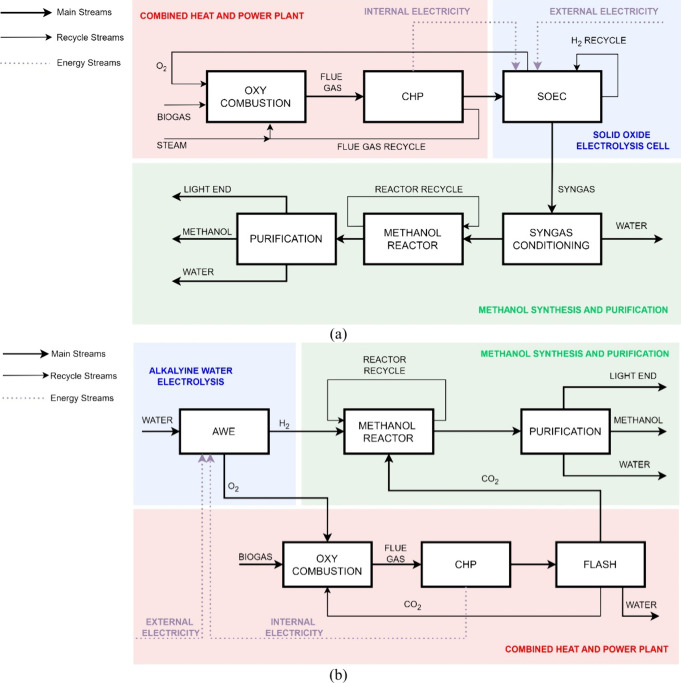
Simplified plant scheme of (a) SOEC route; and (b) AWE route.

The rationale for modeling these two distinct configurations is to systematically compare a highly integrated, high-temperature pathway (SOEC route) with a more mature, low-temperature one (AWE route). This comparison allows for a clear assessment of how the technology choice for hydrogen production impacts overall process performance. Consistently, both processes assume 8000 h/year of operation. In both configurations, heat integration was maximized according to a systematic pinch analysis methodology, whereby the resulting heat exchanger network was refined to avoid an excessive number of heat exchangers and to eliminate impractical stream interconnections between process fluids. The recovered heat is used internally for steam generation and power production; no external heat export was considered. Accordingly, the economic assessment includes methanol revenues and electricity import/export (where applicable), but no revenue stream is assigned to heat. A detailed description of each plant configuration is provided below.

### 2.1 High-Temperature SOEC Route

The SOEC route (Figure 2) integrates a CHP plant and a SOEC coelectrolyzer. The inlet biogas stream enters the plant at a volumetric rate of 2000 Nm^3^/h (stream #S-1 in Figure 2) and is burnt in pure oxygen (stream #S-19), the latter supplied from the SOEC output. Additional steam (stream #S-9, 77.2 kmol/h), internally generated via heat recovery within the CHP section, and a flue gas recycle (stream #S-8, 273.1 kmol/h) are mixed at the inlet of the oxy-combustion to moderate the temperature (1700 °C).^42^ The flue gas and steam recycle streams act as thermal diluents to control the combustion temperature in the oxy-fuel boiler, preventing excessive flame temperatures and ensuring stable operation and efficient downstream heat recovery. The hot flue gas (stream #S-2) then passes through a heat recovery train of heat exchangers, consisting of economizer (HE-3), evaporator (HE-2), and superheater (HE-1) for integration with a steam Rankine cycle. This Rankine cycle is a key component of the CHP system, designed for high-temperature heat recovery. The steam cycle is represented in simplified form to estimate net electricity generation within the process-level assessment. Note that, unlike the AWE route, the SOEC configuration requires the oxy-combustion flue gas to remain available at approximately 800 °C and near-atmospheric pressure as feed to the electrolysis unit; therefore, only the heat above the SOEC operating temperature can be recovered for power generation, making a Rankine cycle an appropriate option for this process configuration. The process under consideration operates with a 100 bar inlet pressure, a steam inlet temperature of 538 °C, and a condenser pressure of 0.07 bar. The driving force for this process is the thermal duty from the oxy-combustion boiler. Stream #S-5, which is set to exit the last heat exchanger at 800 °C, is then partly (49%) sent to the SOEC and partly (51%) recycled for temperature moderation in the boiler. Before being recycled, further heat recovery is achieved by cooling downstream #S-6 in two heat exchangers (LPS-EXCHANGER, cooling down to 236 °C; HE-4, cooling down to 120 °C).

**Figure 2:**
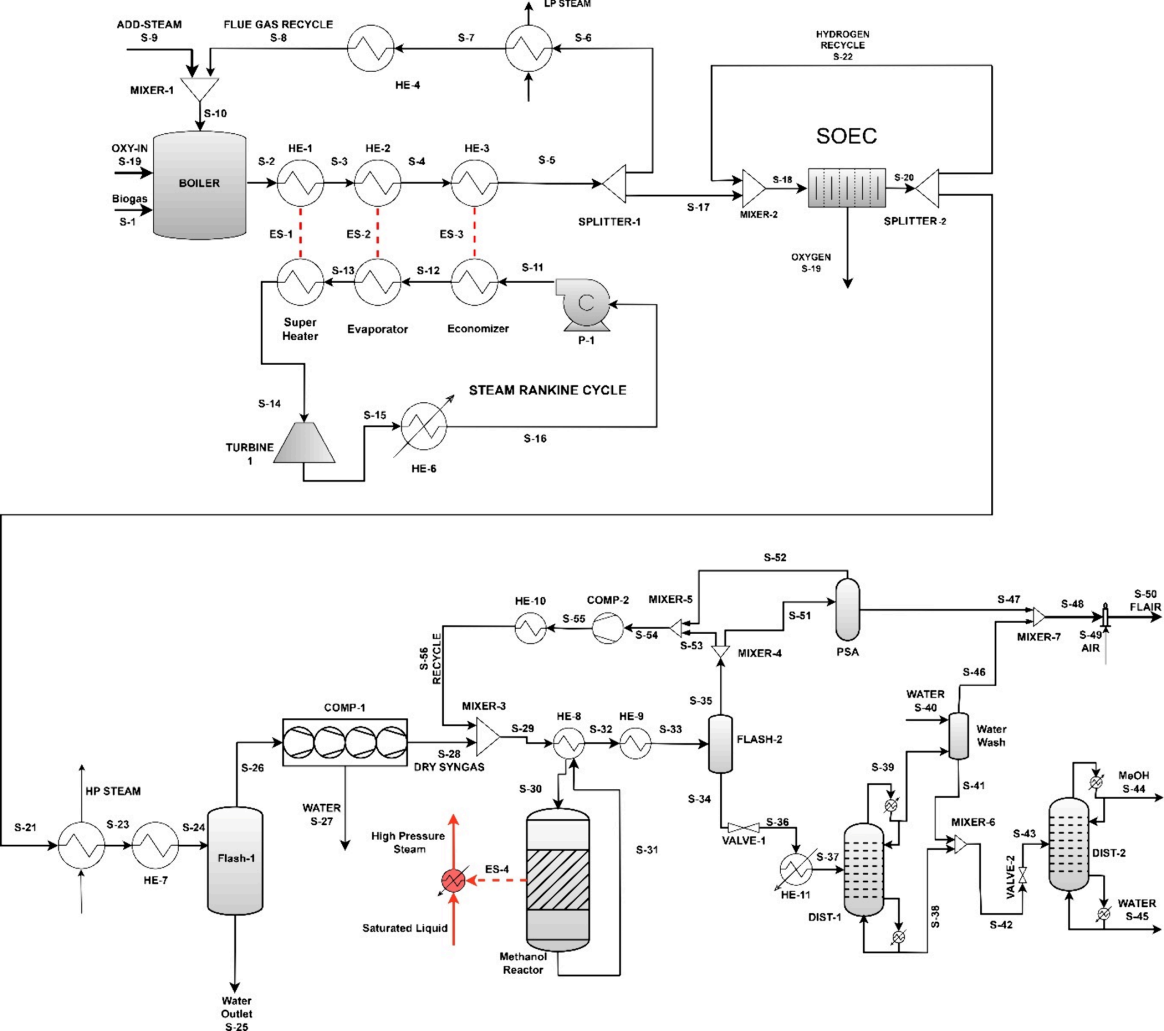
Detailed plant scheme of the SOEC route.

Parallelly, stream #S-17 is mixed with a hydrogen recycle (stream #S-22) before entering the SOEC unit, which operates isothermally at 800 °C. Stream #S-22 (17.8% of the total syngas) is meant to maintain a reducing atmosphere and to prevent cathode degradation.^43^ The SOEC performs coelectrolysis, where steam and carbon dioxide are electrochemically reduced at the cathode, while oxide ions are oxidized at the anode. Overall, the reactions at the cathode side are^44^

$$H_{2}O+2e^{-}\to H_{2}+O^{2-}$$ 1

$$CO_{2}+2e^{-}\to \mathrm{CO}+O^{2-}$$ 2

and at the anode side:

$$2O^{2-}\to O_{2}+4e^{-}$$ 3

The SOEC unit yields two primary products, namely a pure oxygen stream (stream #S-19), which is recycled to the CHP plant, and a hot syngas stream (stream #S-21) with a resulting composition of 56.3%_mol_ H_2_ and 26.1%_mol_ CO. This corresponds to an H_2_/CO ratio of approximately 2.16, which lies within the typical industrial operating range for methanol synthesis (2.0–2.2); the SOEC operating conditions were tuned to approximate this stoichiometric target. The plant then includes a methanol synthesis and purification section. The high-temperature outlet syngas (stream #S-21) from the SOEC unit is cooled down to 275 °C to generate high-pressure steam, then it is further cooled down to 40 °C in two consequent heat exchangers. The first one produces high-pressure steam and also reduces the cooling water consumption of HE-7, which further cools down the stream #S-23. Water is condensed and removed in FLASH-1. The cold and dry syngas (stream #S-26) is compressed to 75 bar in a 3-stage compressor with intercooling, and is further dried before being mixed with a recycle stream #S-56 from the methanol reactor. Stream #S-29 is then preheated by the hot reactor outlet (stream #S-31).

The methanol reactor is modeled as an isothermal multitubular fixed-bed reactor operating at 250 °C and 75 bar, and it is kept at constant temperature via saturated high-pressure steam in the shell side (shown as ES-4 in Figure 2). Process stream pressure drops across the reactor were calculated according to the Ergun correlation. The hot reactor products are cooled down to 40 °C before entering a high-pressure flash drum. The vapor phase from this flash (stream #S-35) is then split, with a small purge sent to a pressure swing adsorption (PSA) unit to recover H_2_, while stream #S-52 is sent to compression (COMP-2) and is then recycled (stream #S-56). The liquid phase from the flash (stream #S-36) is depressurized and fed to HE-11 for preheating from 28 to 54 °C, and then sent to the first distillation column (stream #S-38), which operates at 2.2 bar to separate light products. The overhead vapor from this column (stream #S-39) is sent to a water wash column, where a small quantity of remaining methanol is recovered by absorption into a freshwater stream. The methanol-rich stream from the bottom of the wash column (stream #S-42) is mixed with the bottom products from DIST-1 (stream #S-40) and fed to the second distillation column (stream #S-44), while the cleaned light-end gases are burned together with the byproducts from the PSA. The second column (DIST-2) operates at 1.1 bar to separate water and produce methanol at 99.5%_wt_ purity.

### 2.2 Low-Temperature AWE Route

The detailed plant scheme of the AWE route is presented in Figure 3. The inlet biogas (stream #S-1 in Figure 3) of 2000 N m^3^/h is compressed to 30 bar, then mixed with 71.6 kmol/h of recycled CO_2_ (stream #S-11) and with 120 kmol/h of pure oxygen supplied from the AWE (stream #S-23) before oxy-combustion. The temperature of the hot, high-pressure flue gas exiting the chamber (stream #S-3) is limited to 1250 °C by means of a CO_2_ recycle. This temperature is a critical design constraint set by the maximum allowable inlet temperature at the downstream gas turbine,^45^ where the flue gas (stream #S-3) gets expanded to 10 bar to generate electricity. The hot turbine exhaust (stream #S-4) exits at 1089.5 °C and provides the thermal duty for the bottoming Rankine cycle, operating at 120 bar and designed for a condenser pressure of 0.05 bar. It should be noted that, for comparison, in the SOEC route (Figure 2), since the flue gas does not pass through a turbine, the temperature upper bound for the boiler was set to a higher value (1700 °C). The resulting high-pressure steam (stream #S-14), at 118 bar, expands in the high-pressure (HP) turbine to an intermediate pressure of 16 bar. This steam (stream #S-15) is then reheated with the hot turbine outlet. The reheated steam (stream #S-16) then expands in a low-pressure (LP) turbine down to 0.05 bar. The outlet stream #S-17 is sent to the CONDENSER HE-4 to close the cycle. After providing heat to the Rankine cycle, the main flue gas stream (stream #S-6) is further cooled down to 35 °C in HE-3 and enters a flash drum where water is removed (stream #S-8) and the resulting CO_2_-rich (99%_mol_) gas stream is obtained (stream #S-9, 579.2 kmol/h), being this partly sent to methanol synthesis (stream #S-10), partly recycled to oxy-combustion (stream #S-11).

**Figure 3:**
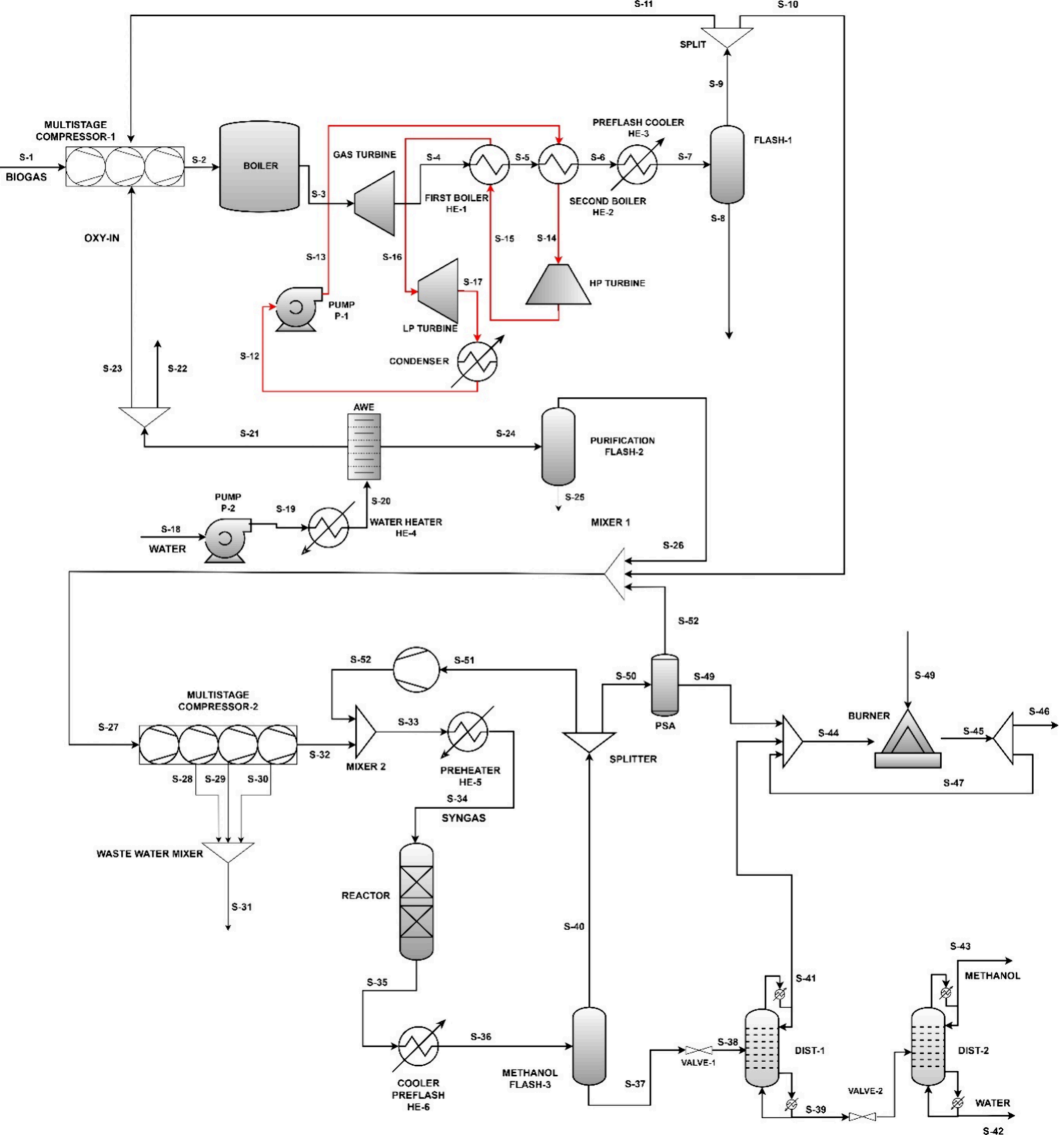
Detailed plant scheme of the AWE route.

The AWE unit has an inlet water flow rate of 243.8 kmol/h (stream #S-18) at ambient conditions, which is pumped to 10 bar. This stream (stream #S-19) is then brought up to 70 °C in WATER HEATER HE-5 and fed to the AWE unit operating at 10 bar and 70 °C. The AWE unit produces 240 kmol/h of H_2_ and 121.6 kmol/h of O_2_. The overall reaction is endothermic (Δ*H* = +286 kJ/mol), and the mechanism at the cathode and anode side is^46^

$$2H_{2}O+2e^{-}\to H_{2}+2OH^{-}$$ 4

$$2OH^{-}\to 1/2O_{2}+H_{2}O+2e^{-}$$ 5

The pure O_2_ gas (stream #S-20) is split into two streams, where 99.2 kmol/h (stream #S-21) is sent to the CHP unit, while the remaining 22.3 kmol/h is assumed to be sold as a byproduct (stream #S-22). The hydrogen produced (stream S-24) is sent to a purification flash drum. Here, any remaining water is condensed and removed (stream S-26), producing the final high-purity hydrogen stream (stream S-27), which is sent to the methanol synthesis section.

The pure H_2_ stream from electrolysis (stream #S-26) is combined with the CO_2_ from the CHP (stream S-10) and a small (5 kmol/h) recycled H_2_ from a downstream PSA unit (stream #S-52). This combined feed (stream #S-27) is then compressed to the synthesis pressure of 75 bar in a four-stage compressor with intercooling. The compressed feed is then mixed with the recycle stream (stream #S-51). For the AWE configuration, the stoichiometric number of the mixed reactor feed (fresh CO_2_–H_2_ plus recycle) was set to approximately 2.1, ensuring consistency with industrial methanol synthesis practice. The resulting stream is preheated in HE-6 and enters a four-bed adiabatic reactor with a Cu/ZnO/Al_2_O_3_ catalyst and intercooling among beds. The inlet temperature to the first bed is set at 220 °C. This difference with respect to the SOEC route (250 °C, isothermal multitubular reactor) reflects the different feed composition, as the SOEC reactor treats a CO/H_2_-rich syngas, while the AWE reactor processes a CO_2_–H_2_ feed where lower temperatures favor CO_2_ conversion at the chosen pressure. The reaction products (stream #S-35) are cooled down to 40 °C in HE-7 and enter the separation and purification section. The liquid phase (stream #S-37), which is the crude methanol product, is sent to the distillation train for purification. The vapor phase (stream #S-40) is mostly recycled back to the reactor as stream #S-51, but a 5% purge (stream #S-50) is sent to a PSA unit. The PSA recovers the small remaining high-purity H_2_ (stream #S-52), which is recycled back to the fresh feed at MIXER 1. The tail gas from the PSA (stream #S-49), containing light ends, is sent to a burner for heat recovery. The purification train consists of two distillation columns.

The crude methanol (stream S-37) is expanded through a valve and fed to the first column. Topping column DIST-1 separates light ends (mostly incondensable gases and dimethyl ether) in its overhead stream (stream S-41), which are sent to the burner. The bottoms product (stream S-39), a mixture of methanol and water, is expanded and fed to the second column DIST-2, where it separates water (stream S-42) as the bottom product. The final methanol product exits at the top of this column (stream S-43) at 99.5%_wt_ purity, consistent with the SOEC route.

## 3 Methods

This section details the methodology employed for process simulations, for carrying out the techno-economic analysis, and for the environmental assessment in terms of the CO_2_ emission performance of the proposed biogas-to-methanol process configurations.

### 3.1 Process Simulation

Both process configurations were simulated in Aspen+ v.14 software. Thermodynamic property models were selected on the basis of process conditions and fluid composition. For high-temperature units (CHP and SOEC electrolysis), the Peng–Robinson–Boston–Mathias equation of state was applied. The methanol synthesis reactors and high-pressure gas handling units in both routes were described by using the Peng–Robinson model. The AWE electrolysis unit, operating with an aqueous KOH solution, was modeled using Electrolyte NRTL. For the methanol purification trains in both configurations, the NRTL-RK model was employed to accurately represent the methanol–water-light ends system.^15,22,47^

The high-temperature SOEC unit was modeled via a hierarchical structure in Aspen Plus (Figure 4), made of multiple blocks that are capable of describing all the phenomena occurring within the cell.^48^ Specifically, the mixture entering the cathode (i.e., the flue gas), after passing through the gas diffusion layer, comes into contact with the nickel catalyst, where, given the high temperatures, the water–gas shift (WGS) reaction occurs rapidly, and thermodynamic equilibrium is reached. This is simulated using a Gibbs reactor, RGIBBS-1. As the reactant mixture diffuses within the cathode, the actual electrolysis takes place, breaking down CO_2_ and H_2_O molecules according to the following stoichiometry:

$$H_{2}O\to H_{2}+0.5O_{2}$$ 6

$$CO_{2}\to \mathrm{CO}+0.5O_{2}$$ 7

**Figure 4:**
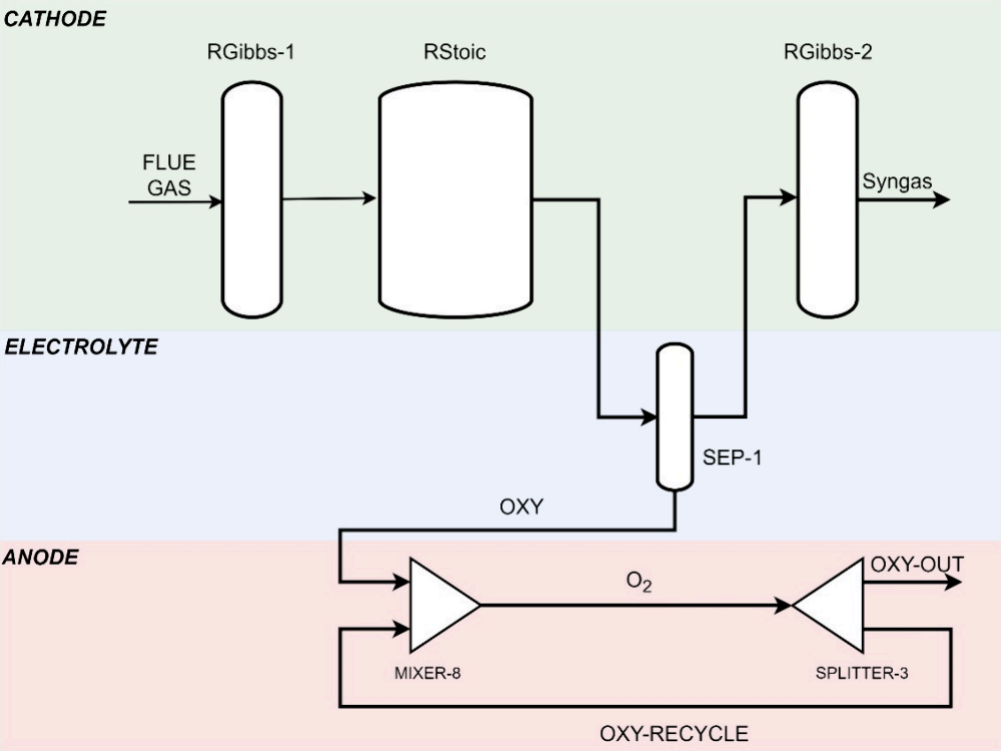
Detailed design of the SOEC unit for process simulation.

This process is simulated using a stoichiometric reactor (Rstoic) where the electrolysis reactions are specified as inputs. Reactions 6 and 7 are described by a specified reactant utilization (RU), which represents the conversion factor of incoming reactants (H_2_O and CO_2_), here assumed equal to 80%.^49^ The oxygen ions produced then diffuse through the solid electrolyte of the membrane and are separated from the reactant mixture, which is modeled by an ideal separator, SEP-1. An oxygen recycle is also simulated within the anode, simulating the sweep gas flow. The remaining gas diffuses toward the cell outlet, encountering the Ni catalyst again, which catalyzes the reattainment of the thermodynamic equilibrium for the WGS reaction, modeled with the Gibbs reactor RGIBBS-2. The cell electric power input was set equal to the net heat duty required by the blocks RGibbs-1, RStoic, and RGibbs-2, in order to maintain the process isothermal at 800 °C.

Differently, the AWE unit was simulated by using a custom model as proposed by Abdin et al.^50^ This model, set to operate at 70 °C and 10 bar with a 35%_wt_ KOH electrolyte, calculates the power consumption by determining the required cell voltage, based on the specified hydrogen production rate (in our case, 240 kmol/h), operating conditions, and internal cell resistances.

The oxy-combustion boiler in both the SOEC and AWE routes was modeled as a stoichiometric reactor (RStoic), assuming complete combustion.

For methanol production, the following set of reactions was considered, including methanol synthesis, the water–gas shift reaction, and the formation of byproducts (ethanol and dimethyl ether):

$$CO_{2}+3H_{2}↔CH_{3}\mathrm{OH}+H_{2}O$$ 8

$$\mathrm{CO}+H_{2}O↔CO_{2}+H_{2}$$ 9

$$2\mathrm{CO}+4H_{2}\to C_{2}H_{5}\mathrm{OH}+H_{2}O$$ 10

$$2CH_{3}\mathrm{OH}\to CH_{3}\mathrm{OC}H_{3}+H_{2}O$$ 11

In particular, reactions 8 and 9 were modeled by using the Langmuir–Hinshelwood–Hougen–Watson rate expressions proposed by Bussche and Froment,^51^ while reactions 10 and 11 representing byproduct formation were modeled using simplified power-law kinetic expressions to provide an approximate estimation of their contribution.^52,53^

The formation of byproducts was preliminarily assessed by considering the full reaction network. For the SOEC route, under the investigated operating conditions, the production of ethanol and dimethyl ether was found to be negligible and did not significantly affect the overall mass balance. Therefore, in this case, reactions 10 and 11 were neglected, and the model was simplified to include only reactions 8 and 9. In contrast, for the AWE route, the formation of byproducts, particularly dimethyl ether, was found to be slightly more pronounced. Although their amounts remain limited, reactions 10 and 11 were retained in the model to account for their contributions.

The two processes are characterized by a different inlet composition: in the SOEC-based route, coelectrolysis directly produces syngas at the optimal ratio for methanol synthesis, while in the AWE-based route, only CO_2_ and H_2_ are present; in this case, CO is generated in situ via the reverse water–gas shift reaction (RWGS), which proceeds in parallel with methanol synthesis. The different feed compositions are also reflected in different choices for the reactor configuration. In the SOEC route, the methanol synthesis step is modeled using an isothermal multitubular fixed-bed reactor (RPFR block) operating at 250 °C and 75 bar. The reactor was sized with 130 tubes, each 1.2 m in length and 4.85 cm in diameter, achieving a single-pass conversion of approximately 7%. In the AWE route, the methanol synthesis is simulated using a sequence of four adiabatic reactor blocks (RPlug) with intercooling, representing a multibed reactor operating at 75 bar. The bed inlet temperatures are set between 220 and 230 °C, achieving a single-pass conversion of approximately 34%. Both configurations employ the same Cu/ZnO/Al_2_O_3_ catalyst, but the reactor design accommodates the different heat management requirements imposed by the feed composition. The syngas composition in the SOEC route enables simple isothermal operation, while in the case of the AWE route, the highly exothermic coupled reactions require a multibed adiabatic configuration with intercooling to maintain catalyst activity. The purification train consists of a Flash separator, a PSA unit, an RStoic block for the burner, and two distillation columns (RadFrac). Full details on the model parameters for the SOEC and AWE routes are reported in the Supporting Information.

### 3.2 Economic and Environmental Evaluation

The economic evaluation of both the SOEC and AWE routes was based on the steady-state simulation results for mass and energy balances and employed the discounted cash flow method to determine the project's profitability over a defined lifespan. The methodology for estimating capital expenditure (CAPEX) and operating expenditure (OPEX) and conducting the final profitability analysis is detailed below.

The capital cost of equipment was estimated using the factorial costing methodology based on the bare module cost (CBM) technique, as detailed by Turton et al.^54^ All costs were escalated to the assessment year (2025) using the Chemical Engineering Plant Cost Index (CEPCI), which is equal to 816 in the reference year. However, this method was not applicable to nonconventional or novel process units. For these, costs were estimated using specific metrics from the literature. In particular, the capital cost for the SOEC stack was estimated based on its electrical power consumption, assuming a specific cost of 520 €/kW,^40^ while the specific cost of the AWE unit was set equal to 304.3 €/kW,^55^ consistent with Krishnan et al.,^56^ who report costs in the range 242 to 388 €/kW. The cost for the oxy-combustion boiler was estimated based on its thermal power, using a specific cost of 238.6 €/kW_th_.^57^ The equipment CAPEX was then calculated by summing up the bare module costs of all units to obtain the Inside Battery Limits (ISBL) investment. The ISBL was subsequently scaled up using standard factorial methods to determine the Fixed Capital Investment (FCI) and finally the total CAPEX (Table 1). The annualized CAPEX was calculated by multiplying the total CAPEX by the capital recovery factor, which resulted in 0.13147 for a 10% discount rate over 15 years.

**Table 1:** Calculation Methodology Used in the CAPEX and OPEX Assessment^a^

Cost Category	Calculation/Basis
outside battery limits (OSBL)	40% of inside battery limits (ISBL)
total direct cost (TDC)	ISBL + OSBL
Indirect Costs (Engineering and contingency)	25% of TDC (15% engineering, 10% contingency)
fixed capital investment (FCI)	TDC + indirect costs
working capital	15% of FCI
start-up costs	10% of FCI
total capital expenditure (CAPEX)	FCI + working capital + start-up costs
variable operating expenditure (OPEX)	biogas at 60/MWh_th_;^58^ electricity at 177.4 €/MWh_el_;^59^ cooling water at 0.50/m^3^;^54^ catalyst prorated over 4 years
fixed operating expenditure	maintenance (3% of FCI), taxes and Insurance (1.5% of FCI), labor and overheads (60% of labor + maintenance)^54^
plant operating hours	8000 h/year

a Full details in the Supporting Information.

The annual OPEX was calculated as the sum of variable expenditures, including biogas feedstock, electricity, and cooling water, as well as catalyst replacement. Fixed OPEX costs were also included to cover maintenance, labor (4.5 times the number of operators and their respected salary calculated following Turton et al.^54^), overheads, taxes, and insurance (Table 1).

$$N_{\mathrm{OL}}=(6.29+31.7P^{2}+0.23N_{\mathrm{np}}{)}^{0.5}$$ 12

where *N*_OL_ is the number of operators per shift, *P* is the number of processing steps involving particulate solids handling, and *N*_np_ is the number of process units that do not involve solids handling. The profitability and scenario analysis were based on a discounted cash flow analysis to calculate the Net Present Value (NPV) over a 15 year lifetime, assuming a 2 year construction phase. Other assumptions are a 48% corporate tax rate and 10 year straight-line depreciation. Finally, the breakeven methanol price is the minimum price of methanol [€/t] at which the NPV of the plant equals zero over the 15 year lifetime.

Biogas production and upgrading are not explicitly modeled, as the system boundary is defined downstream of gas conditioning; however, the adopted biogas price reflects current industrial costs, including production and purification steps. Thus, sensitivity analyses were conducted by varying key economic parameters, including electricity price, biogas price, and the specific capital cost of the electrolysis units; detailed assumptions and numerical results are reported in the Supporting Information.

To quantitatively compare the performance of the SOEC and AWE configurations from a technical, economic, and environmental point of view, several Key Performance Indicators (KPIs) were defined:

- The primary metric for energy consumption is the specific electricity consumption (MWh_el_/t of methanol), which quantifies the net electrical energy imported from the grid per unit of methanol produced. This is calculated as the total electrical power consumed by all process units minus the total power generated by the CHP cycle and then normalized by the mass output of methanol.
- The conversion efficiency of the processes is evaluated via the carbon efficiency (η_carbon_), defined as the percentage of carbon atoms from the biogas feedstock that are ultimately converted into the methanol product. This KPI assesses the effectiveness of the process in utilizing the renewable carbon source.
- The environmental performance was quantified by the carbon intensity (*CI* [t_CO2e_/t of methanol]), used to compare the CO_2_ avoidance performance of the proposed routes and to establish benchmarks against conventional (i.e., fossil-based) methanol production (0.54–0.76 tCO_2e_/t of methanol).^8,15^ *CI* is calculated as follows:
$$\mathrm{CI}=\frac{E_{1}+E_{2}+E_{3}-E_{4}}{\mathrm{methanol}\;\mathrm{production}}$$ 13

Biogas production emissions (*E*_*1*_) represent the CO_2_ emissions associated with biogas generation, accounting for methane leakage during production, process energy consumption, and upstream inefficiencies. On the basis of Scomazzon et al.,^15^ a moderate carbon intensity of 18 gCO_2e_/MJ (ranging between 10 and 27 gCO_2e_/MJ depending on feedstock and technology) was applied to the biogas energy content for the baseline case with 2000 Nm^3^/h biogas flow. In the sensitivity analysis, detailed subsequently, the biogas carbon intensity was assumed to be constant across European countries in order to isolate the impact of electricity grid variability. Direct process emissions (*E*_*2*_) are all those CO_2_ emissions released within the process boundary from the biogenic feedstock; they are here treated as carbon-neutral given the biogenic origin of biogas. Indirect emissions (*E*_*3*_) account for emissions generated outside the process boundaries and are attributable to the plant operation, in particular, the grid electricity consumption. As such, these emissions are calculated by multiplying the net electricity imported from the grid by the carbon intensity factor of the national electricity grid. For the baseline Italian scenario, a carbon intensity of 180 g CO_2e_/kWh was used.^60^ Carbon utilization credits (*E*_*4*_) account for carbon that is chemically fixed in the methanol product and depend on its end-use application. If methanol serves as a chemical feedstock for polymers, formaldehyde, or other chemicals, then a full sequestration credit is applied. This credit equals the carbon content of the methanol product (1.374 kg of CO_2_/kg of methanol, based on stoichiometry). When methanol is burnt as a fuel, the biogenic carbon returns to the atmosphere. Accordingly, no utilization credits are applied in this case.

## 4 Results

The SOEC and AWE routes were evaluated through the following scenarios and case studies:

- Baseline scenario (Italy): evaluates the economic performance based on a methanol baseline price of 884 €/t of methanol^3^ and considers the Italian framework in terms of electricity price (177.4 €/MWh_el_)^59^ and grid carbon intensity (180 g CO_2e_/kWh).^60^
- Breakeven methanol cost analysis: determines the minimum methanol selling price required to achieve a null NPV under Italian baseline conditions, i.e., an electricity price of 177.4 €/MWh_el_,^59^ and a biogas price of 60 €/MWh of biogas.^60^
- Sensitivity analysis: assesses the economic results under variations in the capital cost (€/kW) of the electrolysis units (SOEC and AWE), as these are significant cost drivers and subject to market volatility and different levels of technological maturity. Also, it evaluates the profitability of plants depending on fluctuations in biogas feedstock cost and electricity price, which are important drivers of variable operating expenditures.
- European scenario: expands the analysis to other European countries beyond Italy, according to country-specific data reported in the Supporting Information. The aim here is to evaluate the economic and environmental performance of the proposed plants at different geographic locations.

### 4.1 Technical Performance

This section presents the technical and environmental KPIs obtained from the simulation results with particular emphasis on electricity consumption and plant efficiency (Table 2).

**Table 2:** Summary of Key Energy and Product Methanol Balance for the SOEC and AWE Routes

technical results	SOEC route	AWE route	Unit
**Electric Power Balance**			
CHP power output	–2.4	–4.8	[MW_el_]
electrolyzer	+15.58	+22.53	[MW_el_]
compression	+1.08	+0.59	[MW_el_]
**Net Plant Performance**			
methanol output production	17.5	19.7	[kt/year]
specific electricity consumption	6.49	7.45	[MWh_el_/t of methanol]
carbon efficiency	76.6	88.8	[%]
carbon intensity	0.15	0.32	[t CO_2e_/t of methanol]

The SOEC electrolyzer consumes 44.5% less electric power (15.58 MW_el_) than the AWE unit (22.53 MW_el_) for the same hydrogen output. However, this is partially offset by the more energy-efficient process downstream of the AWE route. In fact, the combined cycle of the AWE route generates roughly double the electric power (4.8 MW_el_ vs 2.4 MW_el_), and its more effective methanol synthesis loop (34% vs 7% single-pass conversion) requires substantially less compression power. Overall, the SOEC route achieves a specific electricity consumption of 6.49 MWh_el_/t of methanol, that is, −13% compared to the AWE route (7.45 MWh_el_/t of methanol). This finding confirms the thermodynamic benefits of high-temperature electrolysis, which provide an advantage in terms of energy demand at the integrated plant scale. The simulation results indicate a fundamental trade-off between carbon utilization and electrical efficiency for the two configurations. The AWE route demonstrates a higher carbon efficiency, converting 88.8% of the biogenic carbon inlet into the final methanol product (19.7 kt/year). This result supports the larger annual methanol production capacity of this route since both routes are characterized by the same biogas inlet. In contrast, the SOEC route achieves a carbon efficiency of 76.6%, resulting in a lower annual output of methanol (17.5 kt/year). This result highlights that while the AWE configuration is more effective at valorizing the biogenic carbon feedstock into the methanol product, the SOEC route exhibits higher efficiency from an overall energy demand standpoint. The carbon intensity CI values of the two routes were evaluated under Italian baseline conditions. The SOEC route achieves a CI of 0.15 t of CO_2e_/t of methanol, while the AWE route reaches 0.32 t of CO_2e_/t of methanol. Both values are significantly lower than the methanol fossil benchmark range (0.54–0.76 t CO_2e_/t of methanol). The CI values presented here assume methanol being used as a chemical feedstock, where the biogenic carbon remains sequestered in downstream products or chemical processes. If methanol were intended to be used as a fuel, an additional 1.374 t of CO_2e_/t of methanol would be added to all the pathways, resulting in 1.52 t of CO_2e_/t of methanol for the SOEC route and 1.70 t of CO_2e_/t of methanol for the AWE route (against 1.91–2.13 t of CO_2e_/t of methanol for the fossil benchmark). Notably, 77% (SOEC) to 82% (AWE) of these emissions are related to electricity production, while biogas production accounts for 23%–18%, respectively.

### 4.2 Economic Results under Baseline Scenario (Italy)

Table 3 summarizes the economic performance of SOEC and AWE routes. The SOEC one requires a total CAPEX of 27.63 M€, which is +6.9% higher than the 25.84 M€ required for the AWE case. This difference is primarily due to the higher SOEC stack cost, compared to more mature alkaline technologies. The values of annualized CAPEX are equal to 3.63 M€/year (SOEC route) and 3.40 M€/year (AWE route).

**Table 3:** Capital Expenditures (CAPEX) and Operating Costs (OPEX) Breakdown

economic results	SOEC	AWE	Unit
**CAPEX Breakdown**			
electrolyzer	8.12	7.29	[M€]
oxy-combustion boiler	2.67	2.09	[M€]
methanol synthesis reactor	0.01	0.29	[M€]
distillation train	0.20	0.18	[M€]
other ISBL equipment	1.30	1.96	[M€]
ISBL total	12.63	11.81	[M€]
OSBL	5.05	4.73	[M€]
total direct cost	17.68	16.54	[M€]
engineering (15% of total direct cost)	2.65	2.48	[M€]
contingency (10% of total direct cost)	1.77	1.65	[M€]
total indirect costs	4.42	4.14	[M€]
fixed capital investment	22.10	20.68	[M€]
working capital (15% of fixed capital investment)	3.32	3.10	[M€]
start-up costs (10% of fixed capital investment)	2.21	2.07	[M€]
**OPEX Breakdown**			
electricity cost	20.15	26.02	[M€/year]
biogas feedstock cost	4.67	4.67	[M€/year]
fixed operating expenditures	2.80	2.69	[M€/year]
consumables and other variable expenditures	0.24	0.22	[M€/year]
**Overall Economic Performance**			
total CAPEX	27.63	25.84	[M€]
total annualized CAPEX	3.63	3.40	[M€/year]
total OPEX	27.86	33.60	[M€/year]
methanol production cost	1799	1879	[€/t of methanol]
net present value	–105.2	–133.4	[M€]

In contrast to capital costs, the OPEX analysis favors the SOEC configuration (Table 3). The annual operating costs are equal to 27.86 M€/year for the SOEC route compared to 33.60 M€/year for the AWE case. Electricity consumption is the main variable cost, accounting for 72.3% of OPEX for SOEC and 78.0% for AWE. Overall, the methanol production cost results in 1799 €/t of methanol for the SOEC route and 1879 €/t of methanol for the AWE case (Figure 5a). Annual revenues from methanol sales total 17.4 M€/year for the AWE route and 15.5 M€/year for the SOEC one. These revenues are insufficient to cover the total annual operating expenses. Consequently, the NPV at the end of the 15 year period is negative for both proposed plant configurations: −133.4 M€ for the AWE route and −105.2 M€ for the SOEC case (Figure 5b). Despite the unprofitability of both cases under baseline conditions, the SOEC route demonstrates a 28.2 M€ higher NPV (i.e., + 21% with respect to the AWE route). This economic advantage is explained by the trade-off between CAPEX and efficiency: although the SOEC route requires an additional +1.79 M€ in initial investment, it generates a specific operating saving with respect to the AWE counterpart. To achieve economic breakeven (NPV = 0) under the Italian baseline electricity price of 177.4 €/MWh, the methanol selling price would need to increase to 1919 €/t for the SOEC route and to 2020 €/t for the AWE case. These minimum selling prices indicate that, in the absence of subsidies, the production cost is approximately double the assumed market reference price, highlighting the critical influence of electricity costs on commercial viability, as shown in the sensitivity analysis over electricity price (Figure 5a).

**Figure 5:**
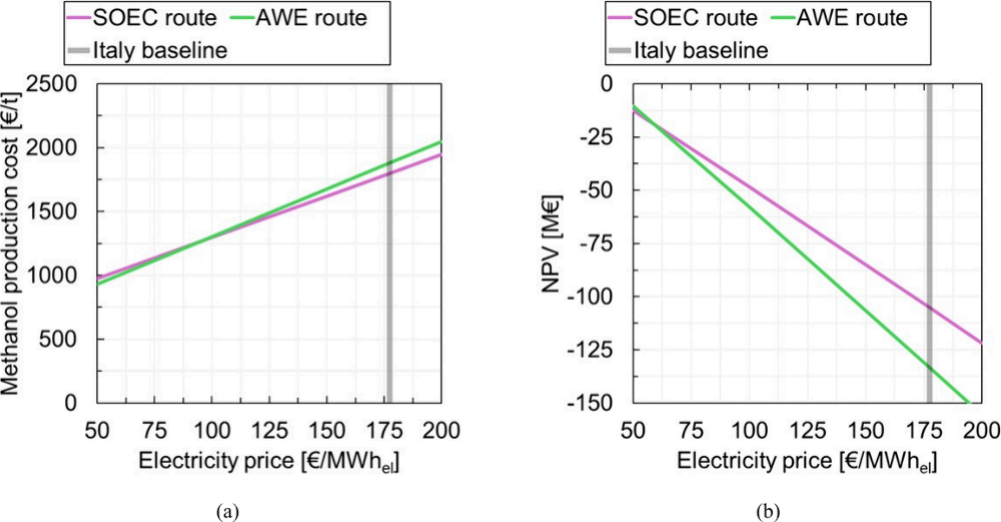
Sensitivity analysis on electricity price [€/MWh_el_] for the Italian base case: (a) methanol production cost [€/t of methanol] and (b) net present value (NPV [M€]).

Sensitivity analyses were conducted on the biogas price, on the capital costs of electrolyzers, and on the electricity price. Varying the biogas price from 30 to 120 €/MWh_el_ (baseline: 60 €/MWh_el_) affects the NPV by approximately 30 M€ for both routes, representing 23–29% of the baseline loss. This confirms that feedstock costs play a minor role compared to other operating expenses. Similarly, variations in the capital cost of electrolyzers show a limited impact on the overall economic performance. A 50% reduction of the capital cost of electrolyzers would improve the NPV by roughly 15–20 M€. The dominance of operating costs over capital investment reinforces the finding that process efficiency and energy pricing are the critical determinants of commercial feasibility. With sensitivity analyses for biogas price and CAPEX confirming their secondary roles, a dedicated sensitivity analysis was performed on the Italian base case to quantify the impact of electricity price, as it is the main driver of operating costs (Figure 5). The results reveal two important findings. First, they expose a technology crossover point at very low electricity prices. While the SOEC route is economically superior in most scenarios due to its 15% higher electrical efficiency, the AWE route becomes the preferable option at electricity prices below approximately 70 €/MWh_el_. Under such a low electricity price regime, the lower initial capital cost of AWE (25.8 M€ against 27.6 M€ for SOEC) becomes a crucial economic factor, especially when considering the differences in methanol production capacities between the two plants. Second, the analysis confirms that even with access to very low-cost electricity, the project remains economically unviable at the reference methanol price of 884 €/t. In fact, at 40 €/MWh_el_, the AWE plant exhibits a still negative NPV (Figure 5b). Also note that we chose a 10% discount rate, assuming a reasonable rate for an investment in a commodity sector. However, for a new technology, the cost of capital is likely to be higher (at least 15–20%), and that would deteriorate the NPV performance even more.

### 4.3 Geographic Sensitivity (Europe)

This section expands the environmental and economic analyses to the broader European context. The environmental and economic viability of the proposed routes is highly sensitive to local grid carbon intensities and electricity prices, respectively, which vary significantly across the 27 European member states. Figure 6 reveals significant geographic variation in the carbon intensity for chemical feedstock applications, showing a strong linear correlation between grid carbon intensity and the methanol production carbon footprint. This outcome demonstrates that the electricity source is the dominant factor in determining the environmental performance. Countries with low-carbon electricity grids achieve carbon-negative methanol production (negative bars), while high-carbon grids result in performance worse than that of the fossil benchmark range. Nordic countries demonstrate the strongest environmental performance because of low-carbon electricity grids. For instance, Sweden (7 g of CO_2e_/kWh grid intensity) achieves carbon intensities of −1.00 t of CO_2e_/t of methanol for AWE and −0.97 t of CO_2e_/t of methanol for SOEC in chemical applications, representing significant improvements over the traditional fossil methanol benchmark.

**Figure 6:**
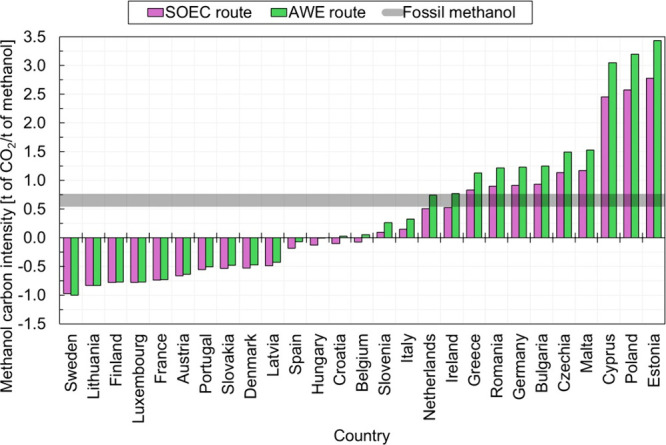
CI across 27 European countries, and comparison with fossil methanol production.

The observed correlation confirms that direct coupling with dedicated renewable electricity (e.g., wind or solar) is the key to maximizing the environmental performance of such plants. In fact, countries with carbon-intensive electric grids show a poor environmental performance. Estonia, with an electric grid carbon intensity of 585 g CO_2e_/kWh, would generate +3.43 t of CO_2e_/t of methanol for AWE and +2.78 t of CO_2e_/t of methanol for SOEC, significantly higher values than the fossil methanol counterpart. The distinction between methanol chemical and fuel use remains an important point. For chemical applications with long-term carbon utilization, the process achieves an 8–192% improvement over the traditional fossil methanol, even when using electricity from the current European grid. Differently, if methanol is deployed as a fuel with consequent re-emission of its embedded carbon content, the environmental benefits of the biogas-to-methanol process strongly depend on the grid carbon intensity.

Still, for the European context, a geographic analysis of electricity prices was conducted to evaluate the profitability of the SOEC and AWE routes. As illustrated in Figure 7, the NPV varies significantly across the European countries. The analysis highlights a vast performance gap between the best and worst cases. Finland, with its low electricity prices (79.7 €/MWh_el_), shows the least negative NPV (−33.5 M€ for SOEC), while Ireland, having the highest electricity costs (263.6 €/MWh_el_), demonstrates the worst performance (−217.6 M€ for AWE). Throughout all countries, SOEC consistently outperforms AWE. The analysis of breakeven methanol prices (Figure 8) further confirms that electricity cost is the key factor for assessing the economic performance of such plants. The breakeven prices range significantly, from 1270 €/t of methanol in Finland (for the AWE route) to 2682 €/t of methanol in Ireland (for the AWE route).

**Figure 7:**
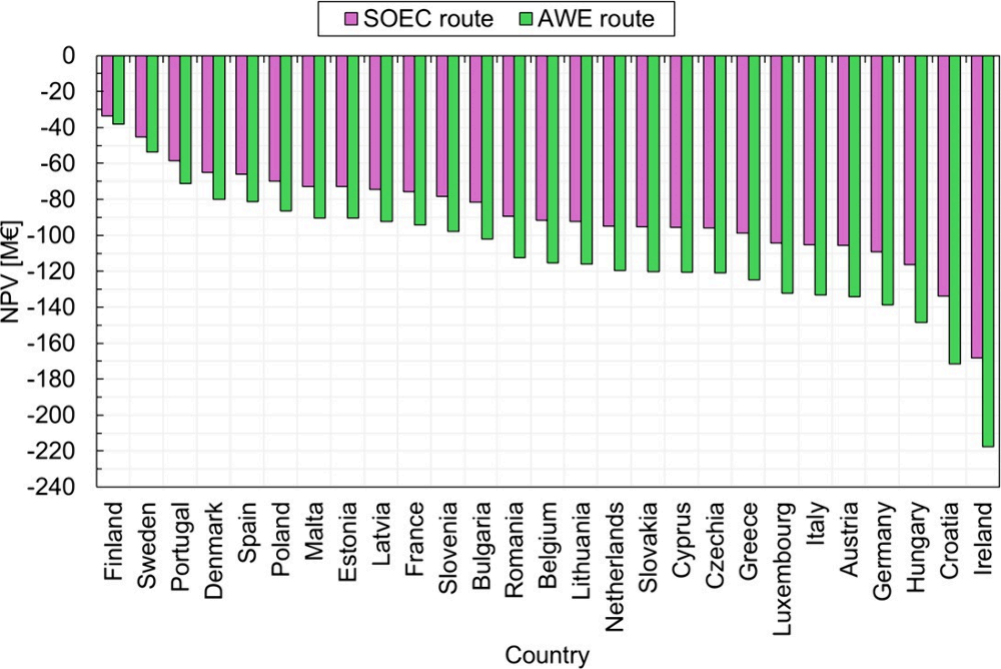
NPV across European countries.

**Figure 8:**
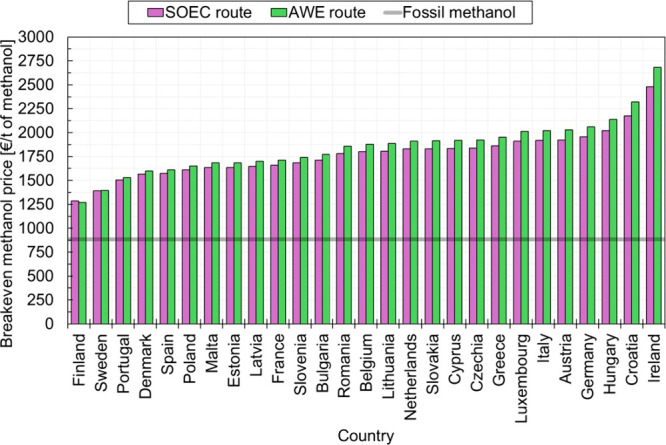
Breakeven methanol price across European countries.

Similarly, a sensitivity analysis was conducted on the methanol selling price. At the baseline 884 €/t of methanol, the plants are economically unviable across all 27 countries. Viability first emerges in Finland at approximately 1270 €/t of methanol (+44% premium), where the AWE route achieves breakeven thanks to the low electricity price (79.7 €/MWh_el_). Economic viability expands more meaningfully beyond 1500 €/t of methanol (+70% premium), where 3 countries (Sweden, Finland, Portugal) achieve positive returns, all characterized by favorable electricity pricing. At 1700 €/t of methanol (+92% premium), viability reaches 11 countries, primarily concentrated in Northern and Southern Europe with competitive electricity markets. Nonetheless, major industrial markets like Germany and Italy remain unprofitable even at this substantial premium, indicating that favorable methanol pricing alone cannot overcome high electricity costs. This analysis confirms that while premium pricing for methanol is essential, low-cost electricity remains the primary determinant of project profitability and geographic competitiveness.

In this view, alternative biogas-to-methanol pathways not relying on electrolysis, such as direct biogas reforming, may represent interesting options to be investigated, with reported levelized cost of methanol spanning between 357 €/t and 1100 €/t,^61,62^ although a fair comparison would require a systematic assessment under the same hypotheses and boundary conditions.

## 5 Conclusions

This study compared solid oxide electrolysis (SOEC)- and alkaline water electrolysis (AWE)-based processes within integrated biogas-to-methanol systems, identifying how technology choice, electricity sourcing, and methanol end-use jointly shape both economic and environmental outcomes.

From a technical standpoint, while the SOEC route offers higher energy efficiency, requiring 15% less electricity per unit of methanol product (6.49 vs 7.45 MWh_el_/t of methanol produced), the AWE route achieves higher carbon efficiency (88.8% against 76.6%), yielding a higher methanol production (19.7 against 17.5 kt/year) from the same biogas feedstock. Economically, both routes remain unprofitable at the fossil-based methanol price of 884 €/t, with net present values ranging from −217.6 M€ (Ireland, AWE route) to −33.5 M€ (Finland, SOEC route). Breakeven methanol prices span from about 1200 €/t of methanol in Finland to about 2500 €/t of methanol in Ireland, reflecting the variation in European electricity prices. Profitability emerges only for significant methanol prices (at least 1700 €/t of methanol). The environmental performance of the SOEC and AWE routes depends almost entirely on local grid carbon intensities. In low-carbon electric grids, biogas-to-methanol can deliver carbon-negative outcomes, reaching about −1 t_CO2e_/t of methanol in Sweden (SOEC) and significantly outperforming the current fossil methanol benchmark (0.54–0.76 t_CO2e_/t of methanol). Oppositely, when installing the plants in countries characterized by a carbon-intensive grid, CO_2_ emissions can exceed the fossil reference, showing that biogas-to-methanol routes only provide environmental benefits when paired with low-carbon electricity. It should be emphasized that the distinction between chemical use of methanol (potentially enabling carbon-negative potential) and its combustion as a fuel (which re-emits the CO_2_ upon combustion) remains an important concept for defining the system boundaries of such analyses.

Overall, the viability of electrolysis-based biogas-to-methanol systems depends on access to low-cost renewable electricity and the development of strong markets for low-carbon fuels. While policy mechanisms such as targeted carbon pricing and accelerated renewable deployment will be essential, the findings of this work show that biogas-to-methanol via SOEC coelectrolysis represents a comparatively promising alternative to low-temperature electrolysis, particularly from an energy-efficienct and economic perspective. In addition, comparison with reforming-based routes can represent an important extension to be considered in future work.

## Supplementary Material



## Data Availability

Data will be made available on request.

## Notes

Detailed process simulation parameters and operating conditions; equipment sizing methodology for heat exchangers, compressors, pumps, reactors, distillation columns, flash vessels, electrolysis units, and CHP systems; capital and operating cost estimation methods and breakdowns; detailed equipment cost data; economic assessment methodology including NPV and breakeven price calculations, geographic and sensitivity analyses; carbon accounting framework and results; stream compositions and thermodynamic properties; list of symbols; supplementary references (PDF)

## Nomenclature

**Acronyms**
AWE: alkaline water electrolyzer
CHP: combined heat and power
KPI: key performance indicator
PSA: pressure swing adsorption
RU: reactant utilization
RWGS: reverse water–gas-shift
SOEC: solid oxide electrolyzer
**Other symbols**
CAPEX: capital expenditure [M€]
CI: carbon intensity [tCO_2_e/t of methanol]
E1: CO_2_ emissions in biogas production [tCO2e]
E2: direct CO_2_ emissions [tCO2e]
E3: indirect CO_2_ emissions [tCO2e]
E4: CO_2_ utilization credit [tCO2e]
FCI: fixed capital investment [M€]
ISBL: inside battery limits [M€]
NPV: net present value [M€]
OPEX: operative expenditure [M€/year]
OSBL: outside battery limits [M€]
TDC: total direct cost [M€]

## References

[1] IPCC . Climate Change 2022 - Mitigation of Climate Change: Working Group III Contribution to the Sixth Assessment Report of the Intergovernmental Panel on Climate Change; Cambridge University Press: Cambridge 10.1017/9781009157926. 2023 (accessed Dec 18, 2025).

[2] IEA . Primary chemicals https://www.iea.org/reports/primary-chemicals. 2023 (accessed Dec 18, 2025).

[3] IRENA . Innovation outlook: Renewable methanol https://www.methanol.org/wp-content/uploads/2020/04/IRENA_Innovation_Renewable_Methanol_2021.pdf. 2021 (accessed Dec 18, 2025).

[4] IEA . World Energy Outlook 2021 https://www.iea.org/reports/world-energy-outlook-2021. 2021 (accessed Dec 18, 2025).

[5] Dalena, F. ; Senatore, A. ; Marino, A. ; Gordano, A. ; Basile, M. ; Basile, A. Methanol production and applications: An overview, in: Basile, A. ; Dalena, F. (eds.). Methanol 2018, 3–28. 10.1016/B978-0-444-63903-5.00001-7.

[6] Bozzano G., Manenti F. (2016). Efficient methanol synthesis: Perspectives,
technologies,
and challenges. Prog. Energy Combust. Sci..

[7] Van-Dal É.S., Bouallou C. (2013). Design and simulation
of a methanol production plant
from CO_2_ hydrogenation. J. Clean.
Prod..

[8] Pérez-Fortes M., Schöneberger J. C., Boulamanti A., Tzimas E. (2016). Methanol synthesis using captured CO_2_ as
raw material: Techno-economic and environmental assessment. Appl. Energy.

[9] Jiang X., Nie X., Guo X., Song C., Chen J. G. (2020). Recent advances
in carbon dioxide hydrogenation to methanol via heterogeneous catalysis. Chem. Rev..

[10] d’
Amore F., Pereira L. M. C., Campanari S., Gazzani M., Romano M. (2023). A novel process for CO_2_ capture from steam methane reformer with molten carbonate fuel cell. Int. J. Hydrogen Energy.

[11] d’
Amore F., Nava A., Colbertaldo P., Visconti C. G., Romano M. C. (2023). Turning CO_2_ from fuel
combustion into e-Fuel? Consider alternative pathways. Energy Convers. Manag..

[12] Liu M., Li C., Koh E. K., Ang Z., Lee Lam J. S. (2019). Is methanol a future
marine fuel for shipping?. J. Phys. Conf..

[13] IMO . IMO strategy on reduction of GHG emissions from ships https://www.imo.org/en/OurWork/Environment/Pages/2023-IMO-Strategy-on-Reduction-of-GHG-Emissions-from-Ships.aspx. 2023 (accessed Dec 18, 2025).

[14] Schorn F., Lohse D., Samsun R. C., Peters R., Stolten D. (2021). The biogas-oxyfuel
process as a carbon source for power-to-fuel synthesis: Enhancing
availability while reducing separation effort. J. CO2 Util..

[15] Scomazzon M., Barbera E., Bezzo F. (2024). Alternative sustainable routes to
methanol production: Techno-economic and environmental assessment. J. Environ. Chem. Eng..

[16] Wang T., Zhou T., Li C., Zhang M., Song Q., Yang H. (2024). Biogas-to-methanol: A new green methanol
production process based
on anaerobic digestion of biomass. Energy Convers.
Manage..

[17] Deutz S., Bardow A. (2021). Life-cycle assessment of an industrial direct air capture
process based on temperature–vacuum swing adsorption. Nat. Energy.

[18] Zhang H., Desideri U. (2020). Techno-economic optimization of power-to-methanol
with
co-electrolysis of CO_2_ and H_2_O in solid-oxide
electrolyzers. Energy.

[19] Van’t Noordende, H. ; van Berkel, F. ; Stodolny, M. Next level solid oxide electrolysis. Institute for Sustainable Process Technology (ISPT) https://ispt.eu/media/20230508-FINAL-SOE-public-report-ISPT.pdf. 2023 (accessed Dec 18, 2025).

[20] Gupta S., Riegraf M., Costa R., Heddrich M. P., Friedrich K. A. (2024). Solid oxide
electrolysis cell-based syngas production and tailoring: A comparative
assessment of co-electrolysis, separate steam, CO_2_ electrolysis,
and steam electrolysis. Ind. Eng. Chem. Res..

[21] Ferrete F., Molina A., Cabello
González G. M., Moreno-Racero Á., Olmedo H., Iranzo A. (2025). Solid oxide
electrolyzers process
integration: A comprehensive review. Processes.

[22] Rajaee F., Guandalini G., Romano M. C., Ritvanen J. (2024). Techno-economic evaluation
of biomass-to-methanol production via circulating fluidized bed gasifier
and solid oxide electrolysis cells: A comparative study. Energy Conv. Manage..

[23] Artz J., Müller T. E., Thenert K., Kleinekorte J., Meys R., Sternberg A., Bardow A., Leitner W. (2018). Sustainable
conversion of carbon dioxide: An integrated review of catalysis and
life cycle assessment. Chem. Rev..

[24] González-Garay A., Frei M. S., Al-Qahtani A., Mondelli C., Guillén-Gosálbez G., Pérez-Ramírez J. (2019). Plant-to-planet analysis of CO_2_-based methanol processes. Energy Environ.
Sci..

[25] Hu K., Fang J., Ai X., Huang D., Zhong Z., Yang X., Wang L. (2022). Comparative
study of alkaline water
electrolysis, proton exchange membrane water electrolysis and solid
oxide electrolysis through multiphysics modeling. Appl. Energy.

[26] Chen C., Yang A. (2021). Power-to-methanol:
The role of process flexibility in the integration
of variable renewable energy into chemical production. Energy Convers. Manag..

[27] Hank C., Gelpke S., Schnabl A., White R. J., Full J., Wiebe N., Smolinka T., Schaadt A., Henning H. M., Hebling C. (2018). Economics & carbon
dioxide avoidance cost of methanol
production based on renewable hydrogen and recycled carbon dioxide
– power-to-methanol. Sustain. Energy
Fuels.

[28] Nguyen T.
B. H., Zondervan E. (2019). Methanol production
from captured CO_2_ using
hydrogenation and reforming technologies: Environmental and economic
evaluation. J. CO2 Util..

[29] Poluzzi A., Guandalini G., d’Amore F., Romano M. C. (2021). The Potential of
Power and Biomass-to-X Systems in the Decarbonization Challenge: a
Critical Review. Curr. Sustain./Renew. Energy
Rep..

[30] Park J., Qi M., Baek J., Choi D., Kwon E. E., Cho H., Lee J. (2025). Bio-e-methanol production via biogas partial oxidation integrated
with solid oxide electrolyzer cell: A comprehensive energy, exergy,
economic, and environmental (4E) analysis. Energy
Convers. Manage..

[31] Detchusananard T., Wiranarongkorn K., Im-orb K. (2025). Assessment of bio-methanol and electricity
co-production via the integration of biomass-to-methanol process,
solid oxide electrolyzer, and power generator. Energy.

[32] Gholizadeh T., Abbaspour N., Ghiasirad H., Skorek-Osikowska A. (2025). Sustainable
biofuel production through anaerobic digestion, SOEC and carbon-capture-and-utilization
(CCU): Techno-economic, exergy and life-cycle analysis. Energy.

[33] Jeong H., Park S., Cho S., Lee I. (2026). Co-utilization
of CO_2_ and biomass in sustainable methanol production:
Enhancing
cost competitiveness and sustainability. Energy
Convers. Manage..

[34] Peng S., Tang Y., Tang J., Deng J., Wang X., Liang X., Huang H., Zheng Z., Ma X. (2025). Techno-economic
analysis and life cycle assessment of renewable methanol production
from MSW integrated with oxy-fuel combustion and solid oxide electrolysis
cell. Int. J. Hydrogen Energy.

[35] Bube S., Sens L., Drawer C., Kaltschmitt M. (2024). Power and
biogas to methanol – A techno-economic analysis of carbon-maximized
green methanol production via two reforming approaches. Energy Convers. Manage..

[36] Pereira F. S., Secchi A. R., Szklo A. (2025). Thermo-Energetic
Analysis of Electrolytic
Oxygen Valorization via Biomass Oxy-Fuel Combustion: A Case Study
Applied to a Power-to-Liquid Route for Methanol Synthesis. Thermo.

[37] Qi M., Alirahmi S. M., Cui C., Zhang Z., Zhang X., Wang L., Chen M., Díaz-Sainz G., Yu H. (2026). Superstructure optimization reveals
novel cost-optimal power-to-methanol
pathways using hybrid solid oxide electrolysis cells. Applied Energy.

[38] Mbatha S., Cui X., Panah P. G., Thomas S., Parkhomenko K., Roger A. C., Louis B., Everson R., Debiagi P., Musyoka N., Langmi H. (2024). Comparative
evaluation of the power-to-methanol
process configurations and assessment of process flexibility. Energy Adv..

[39] Götz M., Lefebvre J., Mörs F., McDaniel Koch A., Graf F., Bajohr S., Reimert R., Kolb T. (2016). Renewable
Power-to-Gas: A technological and economic review. Renew. Energy.

[40] Rajaee F., Romano M. C., Ritvanen J. (2025). Flexible integrated
gasification
solid oxide cell (IGSOC) plant for bio-methanol and bio-power generation. Energy.

[41] EBA . Statistical Report 2025 https://www.europeanbiogas.eu/news/eba-statistical-report-2025/. 2025 (accessed Dec 18, 2025).

[42] Liu Y., Chen S., Yang B., Liu K., Zheng C. (2015). First and
second thermodynamic-law comparison of biogas MILD oxy-fuel combustion
moderated by CO_2_ or H_2_O. Energy Conv. Manage..

[43] Zhou Z., Nadimpalli V. K., Pedersen D. B., Esposito V. (2021). Degradation Mechanisms
of Metal-Supported Solid Oxide Cells and Countermeasures: A Review. Mater..

[44] Zhou H., Cao A., Meng W., Wang D., Li G., Yang S. (2024). Process integration
and analysis of coupling solid oxide electrolysis cell (SOEC) and
CO_2_ to methanol. Energy.

[45] Litrel J., Guillen D. P., McKellar M. (2019). Investigation
of Performance Enhancements
for Air Brayton/ORC Combined Cycles for Small (∼2 MWe) Power
Systems and a Moderate Heat Source Temperature. JOM.

[46] Buttler A., Spliethoff H. (2018). Current status of water electrolysis for energy storage,
grid balancing and sector coupling via power-to-gas and power-to-liquids:
A review. Renew. Sustain. Energy Rev..

[47] Górecki K., Górecka M., Górecki P. (2020). Modelling Properties of an Alkaline
Electrolyser. Energies.

[48] Hauck M., Herrmann S., Spliethoff H. (2017). Simulation of a reversible SOFC with
Aspen Plus. Int. J. Hydrogen Energy.

[49] Wang L., Pérez-Fortes M., Madi H., Diethelm S., herle J. V., Maréchal F. (2018). Optimal design
of solid-oxide electrolyzer based power-to-methane
systems: A comprehensive comparison between steam electrolysis and
co-electrolysis. Appl. Energy.

[50] Abdin Z., Webb C. J., Gray E. M. (2017). Modelling
and simulation of an alkaline
electrolyser cell. Energy.

[51] Bussche K. M. V., Froment G. F. (1996). A steady-state kinetic model for
methanol synthesis
and the water gas shift reaction on a commercial Cu/ZnO/Al_2_O_3_ catalyst. J. Catal..

[52] Chiang C.-L., Lin K.-S. (2017). Preparation and characterization of CuO–Al_2_O_3_ catalyst for dimethyl ether production via methanol
dehydration. Int. J. Hydrogen Energy.

[53] Miranda J. C. C., Ponce G. H. S. F., Arellano-García H., Maciel Filho R., Wolf M. R. M. (2015). Syngas to higher alcohols using Cu-based
catalyst – A simulation approach. Chem.
Eng. Trans..

[54] Turton, R. ; Shaeiwitz, J. A. ; Bhattacharyya, D. ; Whiting, W. B. Analysis, Synthesis, and Design of Chemical Processes; Pearson Education 2018, Fifth.

[55] Yao J., Kraussler M., Benedikt F., Hofbauer H. (2017). Techno-economic assessment
of hydrogen production based on dual fluidized bed biomass steam gasification,
biogas steam reforming, and alkaline water electrolysis processes. Energy Conv. Manage..

[56] Krishnan S., Koning V., Theodorus
de Groot M., de Groot A., Mendoza P. G., Junginger M., Kramer G. J. (2023). Present and future cost of alkaline
and PEM electrolyser stacks. Int. J. Hydrogen
Energy.

[57] NETL . Cost and performance baseline for fossil energy plants volume 1a: Bituminous coal (PC) and natural gas to electricity revision 3 (Report No. DOE/NETL-2015/1723) https://netl.doe.gov/sites/default/files/2020-02/Modular-Staged-Pressurized-Oxy-combustion-Power-Plant-System-Washington-University-in-St.-Louis.pdf. 2015 (accessed Dec 18, 2025).

[58] EBA . Statistical report and market overview https://www.europeanbiogas.eu/wp-content/uploads/2024/12/EBA_stats_report_complete_241204_preview.pdf. 2024 (accessed Dec 18, 2025).

[59] Eurostat . Electricity price statistics-non-household consumers (band IC, excluding taxes and levies) https://ec.europa.eu/eurostat/statistics-explained/index.php?title=Electricity_price_statistics. 2025 (accessed Dec 18, 2025).

[60] EEA . Greenhouse gas emission intensity of electricity generation, country level https://www.eea.europa.eu/en/analysis/indicators/greenhouse-gas-emission-intensity-of-1. 2024 (accessed Dec 18, 2025).

[61] Rinaldi R., Lombardelli G., Gatti M., Visconti C. G., Romano M. C. (2023). Techno-economic
analysis of a biogas-to-methanol process: Study of different process
configurations and conditions. J. Clean. Prod..

[62] Salano L., Bozzini M. M., Moioli E., Manenti F. (2026). Comparative techno-economic
analysis on a biogas reforming plant: Case study on partial upgrading
with a pressure swing water absorption. Chem.
Eng. Sci..

