# Supplementary material for: Techno-Economic Analysis of Biogas-to-Methanol via Integrated Oxy-Combustion and Electrolytic Routes
Source: Ind Eng Chem Res. 2026 Jun 24;65(26):13833–46. doi: 10.1021/acs.iecr.6c00107 (PMC13358967; doi:10.1021/acs.iecr.6c00107)
Supplement: Supplementary file 1 [file ie6c00107_si_001.pdf]

# Techno-economic analysis of biogas-to-methanol via integrated oxy-combustion and electrolytic routes

Mohammad Jafari Mohsen Abad[1,2], Massimiliano Toto[3], Federico d'Amore[1], Elena Barbera[4], Fabrizio Bezzo[1,*]

[1] CAPE-Lab-Computer-Aided Process Engineering Laboratory, Department of Industrial Engineering, University of Padova, via Marzolo 9, Padova, PD 35131, Italy

[2] INSTM – National Interuniversity Consortium of Materials Science and Technology, Via G. Giusti 9, Firenze FI, Italy

[3] Incico S.p.A., via Terranuova, 28, 44121 Ferrara (FE), Italy

[4] BiERLab – Bioprocess Engineering Laboratory, Department of Industrial Engineering, University of Padova, Via Marzolo 9, Padova, 35131, Italy

[*] Corresponding author: fabrizio.bezzo@unipd.it

# Supplementary Material

## Table of Contents

**S1. Process simulation details and model parameters** ........ 3

**S1.1 Process simulation parameters and operating conditions** ........ 3

**S1.2 Equipment Sizing Methodology** ........ 4

S1.2.1 Heat Exchangers ........ 4

S1.2.2 Compressors and Pumps ........ 5

S1.2.3 Methanol Synthesis Reactors ........ 6

S1.2.4 Distillation Columns ........ 6

S1.2.5 Flash Drums and Separation Vessels ........ 6

S1.2.6 Electrolysis Units ........ 7

S1.2.7 Oxy-combustion Boiler and CHP System ........ 7

S1.2.8 Power Generation Equipment ........ 7

**S1.3 Capital Cost Estimation Methodology** ........ 8

S1.3.1 Conventional Equipment Cost Estimation ........ 8

S1.3.2 Novel Equipment Cost Estimation ........ 9

S1.3.3 Total Capital Expenditure (CAPEX) Calculation ........ 9

S1.3.4 Summary of Capital Costs ........ 11

S1.3.5 Major Equipment Cost Breakdown ........ 11

S1.3.6 Cost Distribution Analysis ........ 12
**S1.4 Operating Cost (OPEX) Methodology** ........ 12
S1.4.1 Variable Operating Costs ........ 12
S1.4.2 Fixed Operating Costs ........ 13
S1.4.3 Oxygen By-Product Credit ........ 14
S1.4.4 Summary of Operating Costs ........ 14
S1.4.5 OPEX Distribution Analysis ........ 15
**S2. Detailed Equipment Cost Data** ........ 15
**S3. Economic Assessment Methodology** ........ 18
**S3.1 Net Present Value (NPV) Calculation** ........ 18
S3.1.1 Project Timeline and Depreciation ........ 18
S3.1.2 Annual Cash Flow Calculation ........ 18
S3.1.3 NPV calculation ........ 19
**S3.2 Breakeven Price Calculation** ........ 19
S3.2.1 Breakeven Price Decomposition ........ 19
S3.2.2 Sensitivity Analysis ........ 20
**S3.3 Geographic Analysis Across EU Countries** ........ 21
**S3.4 Sensitivity Analysis Results** ........ 23
**S4. Carbon Accounting Methodology** ........ 24
**S4.1 Carbon Intensity Calculation Framework** ........ 24
**S4.2 Carbon Intensity Results** ........ 26
**S5. Stream properties and composition** ........ 28
**S6. List of symbols** ........ 29
**S7. Supplementary References** ........ 31

# S1. Process simulation details and model parameters

## S1.1 Process simulation parameters and operating conditions

This section provides the key assumptions, operating parameters, and model specifications used in the Aspen Plus V.14 simulations for both process routes described in the main text, namely the Solid Oxide Electrolysis (SOEC) route and the Alkaline Water Electrolysis (AWE) route.

***Table S1.*** *Key unit operation assumptions and parameters (SOEC route).*

| ***Unit Operation*** | ***Parameter*** | ***Value*** | ***Unit*** | ***Notes*** |
|---|---|---|---|---|
| *CHP Plant* | | | | |
| *Boiler* | Model | RStoic | - | Complete $CH_4$ combustion |
| | Max Combustion Temp | 1700 | °C | Controlled via flue gas + steam recycle |
| *Rankine Cycle* | Pressure | 100 | bar | |
| | Turbine Inlet Temp (Steam) | 538 | °C | |
| | Condenser Pressure (Steam) | 0.07 | bar | |
| | Net Power Output | 2.4 | MW | |
| *SOEC Unit* | | | | |
| | Operating Temperature | 800 | °C | Isothermal-thermoneutral voltage |
| | Operating Pressure | 1.013 | bar | |
| | Reactant Utilization (RU) | 80 | % | For $H_2O$ and $CO_2$ |
| | Syngas Recycle Fraction | 17.8 | % | Molar basis, to cathode inlet |
| | Power Consumption | 15.5 | MW | |
| *Methanol Synthesis* | | | | |
| | Reactor Type | Isothermal PFR | - | Multitubular |
| | Operating Temperature | 250 | °C | |
| | Operating Pressure | 75 | bar | Inlet pressure |
| | Reactor Size | 130 tubes: 1.2m L × 0.0485m D | - | |
| | Single-Pass Conversion | ~7 | % | |
| | Recycle Ratio (RR, molar) | 4.5 | - | Reactor recycle / Fresh syngas feed |
| *Purification* | | | | |
| *DIST-1 (Topping)* | 10 stages, 2.2 bar | - | Feed at 5th stage | |
| *DIST-2 (Refining)* | 39 stages, 1.1 bar | - | Feed at 23rd stage | |
| | Methanol Purity Target | 99.5 | % | Distillate of DIST-2 |
| *PSA* | $H_2$ Recovery | 82.5 | % | |
| *Min. Temp Approach* | $\Delta T_{min}$ | 10 | °C | For process-process heat exchangers |

*Note: All simulations were performed in Aspen Plus V.14 with thermodynamic property packages as described in the main text. Operating hours: 8000 h/year.*

***Table S2.*** *Key unit operation assumptions and parameters (AWE route).*

| ***Unit Operation*** | ***Parameter*** | ***Value*** | ***Unit*** | ***Notes*** |
|---|---|---|---|---|
| *CHP Plant* | | | | |
| *Combustor* | Model | RStoic | - | Complete $CH_4$ combustion |
| | Max Combustion Temp | 1250 | °C | Controlled via $CO_2$ recycle |
| *Brayton Cycle* | Pressure | 30 | bar | |
| *Rankine Cycle* | Pressure | 120 | bar | |
| | Condenser Pressure (Steam) | 0.05 | bar | |
| | Net Power Output | 4.8 | MW | |
| *AWE Unit* | | | | |
| | Operating Temperature | 70 | °C | |
| | Operating Pressure | 10 | bar | |
| | Electrolyte | 35 | $\%_{wt}$ KOH | |
| | Power Consumption | 22.5 | MW | |
| *Methanol Synthesis* | | | | |
| | Reactor Type | Adiabatic PFR | - | Multi-bed with intercooling |
| | Beds | 4 | - | With intercooling |
| | Operating Pressure | 75 | bar | Inlet pressure |
| | Total Reactor Volume | 78 | $m^3$ | |
| | Operating Temperature | 220-230 | °C | Bed inlet temperatures |
| | Single-Pass Conversion | ~34 | % | |
| *Purification* | | | | |
| *DIST-1 (Topping)* | 10 stages, 10 bar | - | Feed at 6th stage | |
| *DIST-2 (Refining)* | 22 stages, 1.0 bar | - | Feed at 18th stage | |
| | Methanol Purity Target | 99.5 | % | Distillate of DIST-2 |
| *Min. Temp Approach* | $\Delta T_{min}$ | 10 | °C | For process-process heat exchangers |

*Note: All simulations were performed in Aspen Plus V.14 with thermodynamic property packages as described in the main text. Operating hours: 8000 h/year.*

## S1.2 Equipment Sizing Methodology

The sizing of process equipment was performed based on the steady-state mass and energy balance results obtained from the Aspen Plus V.14 simulations of both routes. Each equipment type was sized according to its characteristic design parameter, with all specific values derived directly from the process simulations. The detailed sizing parameters for each major equipment category are presented below.

### S1.2.1 Heat Exchangers

Heat exchangers were sized based on the required heat duty ($Q$ [W]) and the overall heat transfer coefficient ($U$ [W·m$^{-2}$·K$^{-1}$]). The required heat transfer area ($A$ [m$^2$]) was calculated using:

$$A = \frac{Q}{U \cdot \Delta T_{lm}} \quad \text{(S1)}$$

where $\Delta T_{lm}$ is the log-mean temperature difference (LMTD). The overall heat transfer coefficients used are shown in Table S3.

***Table S3.*** *Overall heat transfer coefficients for different service types.*

| ***Service Type*** | ***U [W·m$^{-2}$·K$^{-1}$]*** | ***Application Example*** |
|---|---|---|
| *Gas-to-gas* | 250 | Flue gas cooling, syngas cooling |
| *Gas-to-water (cooling)* | 600 | General gas cooling with water |
| *Steam-to-liquid* | 1500 | Reboilers with steam heating |
| *Pure component condensation* | 3000 | Water condensation, pure condensers |
| *Mixed vapor condensation* | 1000 | Methanol-water vapor condensation |

**Table S4.** Heat exchanger specifications for SOEC route.

| ***Equipment ID*** | ***Description*** | ***Area [m$^2$]*** | ***Pressure [bar]*** | ***Exchange Type*** |
|---|---|---|---|---|
| *HE-1* | Superheater | 21.43 | 1.013 | Flue gas to steam |
| *HE-2* | Evaporator | 23.54 | 1.013 | Flue gas to steam |
| *HE-3* | Economizer | 6.75 | 1.013 | Flue gas to water |
| *HE-4* | FGR Cooler | 7.69 | 1.013 | Gas-to-steam |
| *HE-5* | FGR Cooler | 29.2 | 1.013 | Gas-to-water |
| *HE-6* | Condenser | 167.1 | 0.07 | Steam condensation |
| *HE-7* | Syngas Cooler | 8.62 | 1.013 | Gas-to-steam |
| *HE-8* | Flue Gas Cooler | 58.9 | 1.013 | Gas-to-water |
| *HE-9* | Reactor F-E HX | 224.8 | 75 | Gas-to-gas |
| *HE-10* | Reactor Cooler | 256.6 | 75 | Gas-to-water |
| *HE-11* | Feed Preheater | 1.02 | 4.76 | Steam-to-liquid |
| *HE-DIST1-CON* | Condenser | 26.5 | 2.2 | Vapor condensation |
| *HE-DIST1-REB* | Reboiler | 5.67 | 4.76 | Steam-to-liquid |
| *HE-DIST2-CON* | Condenser | 49.2 | 1.1 | Vapor condensation |
| *HE-DIST2-REB* | Reboiler | 32.1 | 4.76 | Steam-to-liquid |

***Table S5.*** *Heat exchanger specifications for AWE route.*

| ***Equipment ID*** | ***Description*** | ***Area [m$^2$]*** | ***Pressure [bar]*** | ***Exchange Type*** |
|---|---|---|---|---|
| *HE-1* | Heat Exchanger | 3.1 | 10 | Gas heating |
| *HE-2* | Flue Gas Cooler | 130.7 | 10 | Gas-to-water |
| *HE-3* | $CO_2$/$H_2O$ Cooler | 148.9 | 10 | Gas-to-water |
| *HE-4* | Pure Condenser | 28.8 | 10 | Pure condensation |
| *HE-5* | Steam/Water Heater | 0.593 | 1 | Steam-to-liquid |
| *HE-6* | HP Steam Heater | 92.1 | 40 | Steam-to-gas |
| *HE-7* | HP Gas/Water Cooler | 266.2 | 40 | Gas-to-water |
| *HE-DIST1-CON* | Condenser | 34.0 | 10 | Vapor condensation |
| *HE-DIST1-REB* | Reboiler | 7.4 | 10 | Steam-to-liquid |
| *HE-DIST2-CON* | Condenser | 66.91 | 1 | Vapor condensation |
| *HE-DIST2-REB* | Reboiler | 21.31 | 1 | Steam-to-liquid |

## S1.2.2 Compressors and Pumps

Compressors and pumps were sized based on their electrical power consumption, calculated from thermodynamic principles with assumed efficiencies of 75% for both equipment types.

***Table S6.*** *Compressors and pumps specifications.*

| ***Route*** | ***Equipment*** | ***Power [kW]*** | ***Inlet Pressure [bar]*** | ***Outlet Pressure [bar]*** | ***Function*** |
|---|---|---|---|---|---|
| *SOEC* | Reactor Feed Compressor | 1053 | 1.013 | 75 | Syngas compression |
| *SOEC* | Reactor Recycle Compr. | 28 | 73 | 75 | Recycle compression |
| *SOEC* | Steam Pump | 40 | 0.07 | 100 | Rankine cycle feedwater |
| *AWE* | CHP Compressor | 919 | 1.013 | 30 | Biogas feed compression |
| *AWE* | Reactor Recycle Compr | 9.6 | 74.30 | 75 | Recycle compression |
| *AWE* | Process Pump | 83 | 1 | 10 | Process liquid pumping |
| *AWE* | Water Pump | 4 | 1 | 10 | AWE feed water |

### S1.2.3 Methanol Synthesis Reactors

The SOEC and AWE routes employ different reactor configurations due to different feed compositions and thermal management requirements.

***Table S7.*** *Methanol synthesis reactor specifications.*

| *Parameter* | *SOEC Route* | *AWE Route* |
|---|---|---|
| *Configuration* | Isothermal multi-tubular fixed bed | Multi-bed adiabatic with intercooling |
| *Number of tubes/beds* | 130 tubes | 4 beds |
| *Tube length* | 1.2 m | — |
| *Tube internal diameter* | 0.0485 m (4.85 cm) | — |
| *Total volume (including shell)* | 3.05 $m^3$ | 78 $m^3$ |
| *Operating temperature* | 250°C (constant) | 220-230°C (bed inlets) |
| *Operating pressure* | 75 bar | 75 bar |
| *Catalyst type* | $CuO/ZnO/Al_2O_3$ | $CuO/ZnO/Al_2O_3$ |
| *Catalyst density* | 1775 kg/$m^3$ | 1775 kg/$m^3$ |
| *Catalyst porosity* | 0.4 | 0.4 |
| *GHSV* | 89,000 $h^{-1}$ | 400 $h^{-1}$ |
| *Single-pass conversion* | ~7% | ~34% |
| *Thermal management* | Shell-side steam cooling | Intercooling between beds |

### S1.2.4 Distillation Columns

Distillation columns were designed using rigorous simulations in Aspen Plus to determine stage requirements and were sized based on vapor loading and structural constraints.

***Table S8.*** *Distillation column specifications.*

| *Route* | *Column* | *Diameter [m]* | *Height [m]* | *Stages* | *Pressure [bar]* | *Tray Spacing [m]* | *Function* |
|---|---|---|---|---|---|---|---|
| *SOEC* | DIST-1 | 0.35 | 7.5 | 10 | 2.2 | 0.6 | Light ends removal |
| *SOEC* | DIST-2 | 0.82 | 21.8 | 33 | 1.1 | ~0.66 | Methanol-water separation |
| *AWE* | DIST-1 | 0.31 | 13 | 10 | 10 | ~1.3 | Light ends removal |
| *AWE* | DIST-2 | 0.89 | 24.2 | 22 | 1 | ~1.1 | Methanol-water separation |

*Note: H/D ratios were limited to <30 for structural stability. Product specification: 99.85%$_{wt}$ methanol purity for both routes.*

### S1.2.5 Flash Drums and Separation Vessels

Flash drums were sized based on liquid residence time (5-10 minutes).

***Table S9.*** *Flash drum and separation vessel specifications.*

| *Route* | *Equipment* | *Type* | *Volume [$m^3$] or capacity* | *Pressure [bar]* | *Function* |
|---|---|---|---|---|---|
| *SOEC* | FLASH-1 | Vertical drum | 0.75 | 6 | Water removal from syngas |
| *SOEC* | FLASH-2 | Vertical drum | 0.5 | 15 | Crude methanol separation |
| *SOEC* | WAT-WASH | Packed column | 0.30 m D × 4.0 m H (2.5 m packing) | 6 | Methanol recovery from light ends |
| *AWE* | FLASH-1 | Vertical drum | 0.176 | 10.5 | Water removal from hydrogen |
| *AWE* | FLASH-2 | Vertical drum | 0.461 | 75 | High-pressure V-L separation |
| *AWE* | PSA | Adsorption unit | 5.5 kmol/h capacity | 75 | $H_2$ recovery from purge |

### S1.2.6 Electrolysis Units

The electrolysis units are the key differentiators between the SOEC and AWE routes, with distinct operating principles and electric power requirements.

***Table S10.*** *Electrolysis unit specifications.*

| ***Parameter*** | ***SOEC Route*** | ***AWE Route*** |
|---|---|---|
| *Technology* | Solid Oxide Electrolysis Cell | Alkaline Water Electrolyzer |
| *Electrical power input* | 15580 kW | 22530 kW |
| *Operating temperature* | 800°C | 70°C |
| *Operating pressure* | 1.013 bar | 10 bar |
| *Electrolyte* | Solid oxide (ceramic) | 35 $\%_{wt}$ KOH aqueous |
| *Feed composition* | $CO_2$ + $H_2O$ + 17.8% syngas recycle | Pure water (243.8 kmol/h) |
| *Primary product* | Syngas (56.3% $H_2$, 26.1% CO) | Pure $H_2$ (240 kmol/h) |
| *Secondary product* | Pure $O_2$ for oxy-combustion | $O_2$: 99.2 kmol/h to CHP, 22.3 kmol/h output |
| *Reactant utilization* | 80% (RU) | — |
| *Specific cost* | 520 €/kW [Rajaee et al.[1]] | 304.3 €/kW [Yao et al.[2]] |

*Note: The 17.8% syngas recycle in SOEC maintains a reducing atmosphere to prevent cathode degradation[3].*

### S1.2.7 Oxy-combustion Boiler and CHP System

The oxy-combustion system provides concentrated $CO_2$ while generating thermal energy for power production.

***Table S11.*** *Oxy-combustion boiler specifications.*

| ***Parameter*** | ***SOEC Route*** | ***AWE Route*** |
|---|---|---|
| *Thermal duty* | 11.15 MW | 8.75 MW |
| *Operating pressure* | 1.013 bar | 30 bar |
| *Combustion temperature* | 1700°C | 1250°C |
| *Biogas feed rate* | 2000 $Nm^3$/h | 2000 $Nm^3$/h |
| *Biogas composition* | 60 mol% $CH_4$, 40 mol% $CO_2$ | 60 mol% $CH_4$, 40 mol% $CO_2$ |
| *Oxygen source* | SOEC co-electrolysis | AWE electrolysis (99.2 kmol/h) |
| *Temperature moderation* | 77.2 kmol/h steam + 273.1 kmol/h FGR | 71.6 kmol/h $CO_2$ recycle |
| *Specific cost* | 238.6 €/$kW_{th}$ [NETL[4]] | 238.6 €/$kW_{th}$ [NETL[4]] |

*Note: AWE combustion temperature limited by gas turbine inlet temperature constraint.*

### S1.2.8 Power Generation Equipment

Both routes incorporate thermal-to-electrical energy conversion systems with different configurations.

***Table S12.*** *Power generation equipment specifications.*

| ***Route*** | ***Equipment*** | ***Power [kW]*** | ***Inlet Pressure [bar]*** | ***Outlet Pressure [bar]*** | ***Inlet Temp [°C]*** |
|---|---|---|---|---|---|
| *SOEC* | Steam Turbine | 2355 | 100 | 0.07 | 538 |
| *AWE* | Gas Turbine | 2016 | 30 | 10 | 1250 |
| *AWE* | HP Steam Turbine | 1055 | 120 | 16 | ~550 |
| *AWE* | LP Steam Turbine | 2163 | 16 | 0.05 | ~550 (after reheat) |
| *Total* | SOEC | 2355 | - | - | - |
| *Total* | AWE | 5234 | - | - | - |

*Note: AWE route achieves higher power generation through gas turbine integration and reheat Rankine cycle.*

## S1.3 Capital Cost Estimation Methodology

The capital expenditure (CAPEX) for both process routes was estimated using the factorial bare module cost method developed by Turton et al.[5] This method provides a systematic approach to estimate the total installed cost of process equipment by applying correction factors for operating pressure, material of construction, and installation components (piping, instrumentation, insulation, civil work, etc.).

### S1.3.1 Conventional Equipment Cost Estimation

For conventional process equipment (heat exchangers, pumps, compressors, vessels, columns), the cost estimation follows a four-step procedure:

Step 1: Base Equipment Cost ($C_{p0}$)

The base equipment cost in the reference year 2001 is calculated using a power-law correlation:

$$\log_{10}(C_{p0}) = K_1 + K_2\log_{10}(A) + K_3[\log_{10}(A)]^2 \quad \text{(S2)}$$

where:

- $C_{p0}$ = base equipment cost in $\$^{2001}$;
- $A$ = characteristic size parameter, such as area for heat exchangers, electric power for compressors and pumps, volume for vessels;
- $K_1, K_2, K_3$ = equipment-specific parameters from Turton et al.[5]

Step 2: Pressure Factor ($F_p$)

For equipment operating above atmospheric pressure, a pressure correction factor is applied:

$$\log_{10}(F_p) = C_1 + C_2\log_{10}(P) + C_3[\log_{10}(P)]^2 \quad \text{(S3)}$$

where:

- $P$ = operating pressure in barg (gauge pressure);
- $C_1, C_2, C_3$ = equipment-specific parameters from Turton et al.[5]
- For $P \leq 1$ barg: $F_p = 1.0$.

Step 3: Material Factor ($F_m$)

The material factor $F_m$ accounts for the cost difference between carbon steel (base case) and other materials of construction.

***Table S13.*** *Material factors for major equipment types.*

| *Material of Construction* | *Symbol* | *Heat Exchangers* | *Vessels/Columns* | *Pumps/Compressors* |
|---|---|---|---|---|
| *Carbon Steel (CS)* | $F_m$ | 1.0 | 1.0 | 1.0 |
| *Stainless Steel (SS)* | $F_m$ | 2.0-3.1 | 3.1 | 2.3-2.7 |
| *Nickel Alloys* | $F_m$ | 4.4-7.1 | 7.1 | 4.4-4.7 |

*Note: Specific $F_m$ values depend on equipment type and configuration. All values from Turton et al.*[5]

Step 4: Bare Module Cost ($C_{BM}$)

The bare module cost includes the base equipment cost plus all direct and indirect installation costs:

$$C_{BM} = C_{p0} \cdot F_p \cdot F_m \cdot F_{BM} \quad \text{(S4)}$$

where $F_{BM}$ is the bare module factor that accounts for:

- equipment installation labor;
- instrumentation and controls;
- piping (including valves and fittings);
- electrical systems;
- insulation;
- paint and coatings;
- civil work (foundations, supports).

Typical $F_{BM}$ values range from 1.5-4.0 depending on equipment complexity.

Step 5: CEPCI Escalation to 2025

All costs are escalated from the reference year (2001) to the assessment year (2025):

$$C_{2025} = C_{2001} \cdot \frac{CEPCI_{2025}}{CEPCI_{2001}} = C_{2001} \cdot \frac{816}{397} = C_{2001} \cdot 2.055 \quad \text{(S5)}$$

## S1.3.2 Novel Equipment Cost Estimation

For novel or emerging technology components not adequately represented in standard cost correlations, literature-based specific costs were used.

***Table S14.*** *Specific costs for novel equipment.*

| *Equipment* | *Sizing Parameter* | *Specific Cost* | *Source* | *SOEC Route* | *AWE Route* |
|---|---|---|---|---|---|
| *SOEC Stack* | Electrical power | 520 €/kW$_{el}$ | Rajaee et al.[1] | 15580 kW · 520 €/kW = 8.10 M€ | - |
| *AWE Unit* | Electrical power | 304.3 €/kW$_{el}$ | Yao et al.[2] | - | 22530 kW · 304.3 €/kW = 6.86 M€ |
| *Oxy-combustion Boiler* | Thermal duty | 238.6 €/kW$_{th}$ | NETL[4] | 11150 kW · 238.6 €/kW = 2.66 M€ | 8750 kW · 238.6 €/kW = 2.09 M€ |

*Note: These costs reflect 2025 values and include stack/module costs but exclude balance of plant (BOP) components, which are captured in the OSBL estimate.*

## S1.3.3 Total Capital Expenditure (CAPEX) Calculation

The total CAPEX is built up systematically from equipment costs through several steps.

Step 1: Inside Battery Limits (*ISBL*)

$$ISBL = \sum_{i=1}^{n} C_{BM,i} \quad \text{(S6)}$$

The *ISBL* represents the sum of all bare module costs $C_{BM,i}$ of Eq. (S4) for process equipment $i$.

Step 2: Outside Battery Limits (*OSBL*)

$$OSBL = 0.40 \cdot ISBL \tag{S7}$$

*OSBL* includes:

- utilities infrastructure (cooling water systems, steam generation, electrical distribution);
- site preparation and civil works;
- storage facilities;
- auxiliary buildings (control room, maintenance shop, laboratories);
- environmental protection systems;
- fire protection systems.

The 40% factor of Eq.(S7) is typical for chemical plants with moderate complexity.[5]

Step 3: Total Direct Cost (*TDC*)

$$TDC = ISBL + OSBL \tag{S8}$$

Step 4: Indirect Costs

Indirect costs include engineering, procurement, construction management, and contingency:

$$Engineering = 0.15 \cdot TDC \tag{S9}$$

$$Contingency = 0.10 \cdot TDC \tag{S10}$$

$$Total\ Indirect\ Costs = 0.25 \cdot TDC \tag{S11}$$

The 15% engineering factor covers detailed design, procurement specifications, and construction management. The 10% contingency accounts for unforeseen costs and design changes.

Step 5: Fixed Capital Investment (*FCI*)

$$FCI = TDC + Total\ Indirect\ Costs \tag{S12}$$

Step 6: Working Capital and Start-up Costs

$$Working\ Capital = 0.15 \cdot FCI \tag{S13}$$

$$Start\text{-}up\ Costs = 0.10 \cdot FCI \tag{S14}$$

Working capital covers raw materials inventory, product inventory, and accounts receivable. Start-up costs include commissioning, operator training, and initial performance testing.

Step 7: Total CAPEX

$$CAPEX = FCI + Working\ Capital + Start\text{-}up\ Costs \tag{S15}$$

## S1.3.4 Summary of Capital Costs

***Table S15.*** *Capital cost buildup for SOEC and AWE routes (all values in €$^{2025}$).*

| ***Cost Component*** | ***Calculation Basis*** | ***SOEC Route [M€]*** | ***AWE Route [M€]*** |
|---|---|---:|---:|
| *Inside Battery Limits (ISBL)* | Σ Bare module costs | 12.63 | 11.81 |
| *Outside Battery Limits (OSBL)* | 40% ISBL | 5.05 | 4.73 |
| *Total Direct Cost (TDC)* | ISBL + OSBL | 17.68 | 16.54 |
| *Engineering* | 15% TDC | 2.65 | 2.48 |
| *Contingency* | 10% TDC | 1.77 | 1.65 |
| *Total Indirect Costs* | Engineering + Contingency | 4.42 | 4.14 |
| *Fixed Capital Investment (FCI)* | TDC + Indirect | 22.10 | 20.68 |
| *Working Capital (WC)* | 15% FCI | 3.32 | 3.10 |
| *Start-Up Costs* | 10% FCI | 2.21 | 2.07 |
| *Total Capex* | FCI + WC + Start-up | 27.63 | 25.84 |

*Note: SOEC route has higher CAPEX than AWE route. Equipment costs estimated using Turton et al.[5] methodology with CEPCI 2025 = 816. Novel equipment (SOEC stack, AWE unit, oxy-combustion boiler) costs from literature sources. Currency conversion: USD to EUR at 0.92 exchange rate.*

## S1.3.5 Major Equipment Cost Breakdown

***Table S16.*** *Top 10 equipment costs for SOEC route (€$^{2025}$, ranked by bare module cost).*

| ***Rank*** | ***Equipment*** | ***Type*** | ***Size Parameter*** | ***$C_{BM}$ [M€]*** | ***% of ISBL*** |
|---|---|---|---|---:|---:|
| *1* | SOEC Stack | Electrolyzer | 15580 kW | 8.12 | 64.3% |
| *2* | Oxy-combustion Boiler | Fired Heater | 11.15 MW | 2.67 | 21.1% |
| *3* | Steam Turbine | Turbine | 2355 kW | 0.32 | 2.5% |
| *4* | Reactor Feed Compressor | Compressor | 1053 kW | 0.25 | 2.0% |
| *5* | DIST-2 | Column | 0.82m × 21.8m | 0.17 | 1.4% |
| *6* | HE-10 | Heat Exchanger | 256.6 m² | 0.12 | 1.0% |
| *7* | HE-9 | Heat Exchanger | 224.8 m² | 0.11 | 0.9% |
| *8* | HE-6 | Heat Exchanger | 167.1 m² | 0.09 | 0.7% |
| *9* | HE-11 | Heat Exchanger | 1.02 m² | 0.06 | 0.5% |
| *10* | HE-8 | Heat Exchanger | 58.9 m² | 0.06 | 0.5% |
| - | Top 10 Total | - | - | 11.99 | 94.9% |
| - | All Equipment Total (ISBL) | - | - | 12.63 | 100.0% |

*Note: The SOEC stack and oxy-combustion boiler alone account for 85.4% of total equipment cost.*

***Table S17.*** *Top 10 equipment costs for AWE route (€$^{2025}$, ranked by bare module cost).*

| ***Rank*** | ***Equipment*** | ***Type*** | ***Size Parameter*** | ***$C_{BM}$ [M€]*** | ***% of ISBL*** |
|---|---|---|---|---:|---:|
| *1* | AWE Unit | Electrolyzer | 22530 kW | 7.29 | 61.7% |
| *2* | Combustion Chamber | Fired Heater | 8.75 MW | 2.09 | 17.7% |
| *3* | LP Steam Turbine | Turbine | 2163 kW | 0.31 | 2.6% |
| *4* | Gas Turbine | Turbine | 2016 kW | 0.31 | 2.6% |
| *5* | Reactor | Fixed Bed | 78 m³ | 0.29 | 2.5% |
| *6* | HP Steam Turbine | Turbine | 1055 kW | 0.25 | 2.1% |
| *7* | Comp CHP | Compressor | 919 kW | 0.24 | 2.0% |
| *8* | DIST-2 | Column | 0.89m × 24.2m | 0.15 | 1.2% |
| *9* | HE-7 | Heat Exchanger | 266.2 m² | 0.12 | 1.0% |
| *10* | HE-3 | Heat Exchanger | 148.9 m² | 0.08 | 0.7% |
| - | Top 10 Total | - | - | 11.08 | 94.1% |
| - | All Equipment Total (ISBL) | - | - | 11.77 | 100.0% |

*Note: The AWE unit and combustion chamber account for 79.7% of total equipment cost. The AWE route shows more distributed costs across power generation equipment.*

### S1.3.6 Cost Distribution Analysis

***Table S18.*** *Equipment cost distribution by category.*

| *Equipment Category* | *SOEC Route [M€]* | *% of ISBL* | *AWE Route [M€]* | *% of ISBL* |
|---|---|---|---|---|
| *Electrolysis (SOEC/AWE)* | 8.12 | 64.3% | 7.29 | 61.7% |
| *Oxy-Combustion Boiler* | 2.67 | 21.1% | 2.09 | 17.7% |
| *Heat Exchangers* | 1.01 | 8.0% | 0.76 | 6.4% |
| *Compressors and Pumps* | 0.29 | 2.3% | 0.28 | 2.4% |
| *Turbines* | 0.32 | 2.5% | 0.87 | 7.3% |
| *Reactors* | 0.01 | 0.1% | 0.29 | 2.5% |
| *Separation Equipment* | 0.21 | 1.7% | 0.24 | 2.0% |
| *Total ISBL* | 12.63 | 100% | 11.81 | 100.0% |

*Note: Electrolysis and oxy-combustion equipment dominate costs in both routes. The AWE route has higher turbine costs due to the gas turbine and dual steam turbine configuration, while the SOEC route has significantly higher heat exchanger costs due to extensive thermal integration requirements.*

## S1.4 Operating Cost (OPEX) Methodology

The annual operating expenditure (OPEX) for SOEC and AWE routes was calculated following the methodology outlined in Turton et al.[5], with costs subdivided into variable OPEX costs (dependent on production rate) and fixed OPEX costs (independent of production rate). All operating cost calculations assume 8000 hours/year of operation.

### S1.4.1 Variable Operating Costs

Variable operating costs increase proportionally with methanol production rate and include utilities, raw materials, and operating supplies.

Electricity Cost

As shown in the main text, the electricity consumption represents the main operating cost for both routes. The net electrical power demand was calculated as:

$$P_{net} = P_{electrolysis} + P_{compressors} + P_{pumps} + P_{auxiliaries} - P_{turbines} \quad \text{(S16)}$$

The annual electricity consumption and cost were calculated as:

$$E_{annual} = P_{net} \cdot h_{operating} \quad \text{(S17)}$$

$$C_{electricity} = E_{annual} \cdot c_{electricity} \quad \text{(S18)}$$

where $c_{electricity}$ is the electricity price [€/MWh] and $h_{operating}$ = 8000 h/year.

***Table S19.*** *Annual electricity consumption for both routes.*

| *Parameter* | *AWE Route* | *SOEC Route* | *Unit* |
|---|---|---|---|
| *Annual Net Electricity Consumption* | 146691 | 113575 | MWh/year |
| *Electricity Price (Italy)* | 177.4 | 177.4 | €/MWh |
| *Annual Electricity Cost* | 26.02 | 20.15 | M€/year |

*Note: Electricity price based on Italian industrial tariffs for 2025. Net consumption includes electrolysis, compression, pumping, and auxiliaries minus power generation from turbines.*

Biogas Cost

Both routes have an input of 2000 $Nm^3/h$ of biogas with identical composition (60 mol% CH4, 40 mol% $CO_2$). The annual biogas consumption is:

$$\dot{V}_{biogas,annual} = 2000\ \text{Nm}^3/\text{h} \cdot 8000\ \text{h/year} = 16000000\ \text{Nm}^3/\text{year} \quad \text{(S19)}$$

Converting to energy basis using biogas LHV = 17.5 $MJ/Nm^3$:

$$E_{biogas,annual} = 16000000 \cdot \frac{17.5}{3.6} = 77778\ \text{MWh/year} \quad \text{(S20)}$$

$$C_{biogas} = E_{biogas,annual} \cdot c_{biogas} \quad \text{(S21)}$$

where $c_{biogas}$ = 60 €/MWh is the assumed biogas gate price. Therefore, the annual biogas cost is equal to 77778 MWh/year · 60 €/MWh, giving 4.67 M€/year (both SOEC and AWE routes).

Operating Supplies

Operating supplies include catalysts, chemicals, lubricants, and consumables. These are estimated as:

$$C_{supplies} = 0.10 \cdot C_{maintenance} \quad \text{(S22)}$$

The 10% factor of Eq.(S22) is a standard value for industrial estimates.[5]

### S1.4.2 Fixed Operating Costs

Fixed OPEX costs remain constant regardless of production rate and include labor, maintenance, insurance, taxes, and overhead.

Labor and Supervision

The plant is assumed to require continuous operation with 17 workers covering all shifts, maintenance, and administrative functions. The labor cost is:

$$C_{labor} = N_{workers} \cdot w_{annual} \cdot f_{supervision} \quad \text{(S23)}$$

where:

- $N_{workers}$ = 17 workers
- $w_{annual}$ = 45000 €/worker/year (base salary)
- $f_{supervision}$ = 1.15 (15% premium for supervisory personnel)

Accordingly, the annual labor and supervision cost is equal to 0.88 M€/year for SOEC and AWE routes.

Maintenance, Taxes, and Insurance

Annual maintenance, taxes, and insurance $C_{MTI}$ are estimated as a percentage of *FCI*:

$$C_{MTI} = 0.06 \cdot FCI \quad \text{(S24)}$$

This 6% factor of Eq.(S24) includes:

- routine maintenance and repairs (3%);
- property taxes (1.5%);
- insurance premiums (1.5%).

General Plant Overhead and Administration

General overhead covers administrative staff, utilities for buildings, safety programs, and other plant-level expenses, and is calculated as a fixed 20% of labor ($C_{labor}$) and maintenance costs ($C_{MTI}$):

$$C_{overhead} = 0.20 \cdot (C_{labor} + C_{MTI}) \tag{S25}$$

### S1.4.3 Oxygen By-Product Credit

Both routes produce oxygen as a co-product from electrolysis. However, its economic evaluation is different. As for the SOEC route, all oxygen produced (from co-electrolysis) is internally consumed in the oxy-combustion boiler. As such, no oxygen is available for sale. Different, for the AWE route, oxygen for export is evaluated as follows. Given the total oxygen production of 121.6 kmol/h, of which:

- consumed in oxy-combustion: 99.2 kmol/h;
- available for sale: 22.3 kmol/h, corresponding to 5709 t/year;

the resulting oxygen credit is calculated assuming a 50 €/t market price, accordingly:

$$Revenue_{O_2} = 5709 \text{ t/year} \cdot 50 \text{ €/t} = 285450 \text{ €/year} \approx 0.29 \text{ M€/year} \tag{S26}$$

Oxygen export is evaluated assuming the electrolyser outlet pressure (10 bar); additional drying/compression to meet site-specific delivery specifications is not explicitly modelled.

### S1.4.4 Summary of Operating Costs

***Table S20.*** *Operating cost assumptions and calculated OPEX.*

| ***Parameter*** | ***Unit*** | ***Value*** | ***Source/Assumption*** |
|---|---|---|---|
| *Annual Operating Hours* | h/year | 8000 | User-specified |
| *Electricity Price* | €/MWh | 177.4 | Italian industrial tariff, 2025 |
| *Biogas Price* | €/MWh | 60 | Typical biogas gate price |
| *Worker Salary* | €/worker/year | 45000 | Italian chemical industry standard |
| *Number Of Workers* | workers | 17 | Estimated for plant size |
| *Supervision Factor* | — | 1.15 | 15% premium for supervisors |
| *Maintenance + Tax + Insurance (MTI)* | % of FCI | 6% | Turton et al. (2018) |
| *General Overhead* | % of labor + MTI | 20% | Turton et al. (2018) |

***Table S21***. *Annual operating costs breakdown [M€/year].*

| ***Opex Component*** | ***AWE Route [M€/year]*** | ***SOEC Route [M€/year]*** | ***Calculation Basis*** |
|---|---|---|---|
| *I. Variable OPEX Costs* | | | |
| *Electricity* | 26.02 | 20.15 | Net consumption · 177.4 €/MWh |
| *Biogas* | 4.67 | 4.67 | 77778 MWh/year · 60 €/MWh |
| *Operating Supplies* | 0.22 | 0.24 | 10% of maintenance |
| *Subtotal Variable* | 30.91 | 25.06 | |
| *II. Fixed OPEX Costs* | | | |
| *Labor And Supervision* | 0.88 | 0.88 | 17 workers · 45000 · 1.15 |
| *Maintenance, Tax, Insurance* | 1.35 | 1.33 | 6% of FCI |
| *General Overhead* | 0.45 | 0.44 | 20% of (labor + MTI) |
| *Subtotal Fixed* | 2.68 | 2.65 | |
| *Total OPEX* | 33.60 | 27.86 | |
| *OPEX per ton methanol* | 1707 €/t | 1592 €/t | OPEX / annual production |

### S1.4.5 OPEX Distribution Analysis

***Table S22.*** *Operating cost distribution by category.*

| ***Cost Category*** | ***AWE Route [M€/year]*** | ***% of OPEX*** | ***SOEC Route [M€/year]*** | ***% of OPEX*** |
|---|---|---|---|---|
| *Electricity* | 26.02 | 77.4% | 20.15 | 72.7% |
| *Biogas* | 4.67 | 13.9% | 4.67 | 16.8% |
| *Maintenance, Tax, Insurance* | 1.35 | 4.0% | 1.33 | 4.8% |
| *Labor And Supervision* | 0.88 | 2.6% | 0.88 | 3.2% |
| *General Overhead* | 0.45 | 1.3% | 0.44 | 1.6% |
| *Operating Supplies* | 0.22 | 0.6% | 0.24 | 0.9% |
| *Total OPEX* | 33.60 | 100.0% | 27.86 | 100.0% |

## S2. Detailed Equipment Cost Data

This section provides the complete equipment list and cost breakdown for both SOEC and AWE process routes, ensuring full transparency and reproducibility of the capital cost estimates presented in the main text.

***Table S23.*** *Complete equipment list and cost breakdown for SOEC route.*

| ***Equipment*** | ***Type*** | ***Size*** | ***Unit*** | ***Pressure [bar]*** | ***Base Cost $C_{p0}$ [$]*** | $F_p$ | $F_m$ | ***Bare Module Cost $C_{BM}$ [€]*** |
|---|---|---|---|---|---|---|---|---|
| *SOEC Stack* | SOEC Cell | 15580 | kW | 1.013 | 8830744 | 1.00 | 1.00 | 8124285 |
| *Oxy-Comb. Boiler* | Fired Heater | 11.15 | MW | 1.013 | 2899825 | 1.00 | 1.00 | 2667839 |
| *Steam Turbine* | Steam Turbine | 2354.71 | kW | 100 | 347304 | 1.00 | 1.00 | 319520 |
| *Steam Pump* | Pump | 40 | kW | 100 | 7410 | 2.45 | 1.00 | 35416 |
| *Reactor Feed Comp.* | Compressor | 1053 | kW | 75 | 271896 | 1.00 | 2.00 | 250145 |
| *Reactor Recycle Comp.* | Compressor | 28 | kW | 75 | 26108 | 1.00 | 2.00 | 24019 |
| *Methanol Synthesis Reactor* | Fixed Bed Reactor | 130 | $m^3$ | 75 | 1934 | 1.00 | 2.00 | 10479 |
| *Flash-1* | Vertical Flash Drum | 0.75 | $m^3$ | 6 | 2774 | 1.00 | 1.00 | 10385 |
| *Flash-2* | Vertical Flash Drum | 0.5 | $m^3$ | 15 | 2356 | 1.00 | 1.00 | 8821 |
| *Wat-Wash* | Packed Column | 0.30×4.0×2.5 | $m^3$ | 6 | 1922 | 1.00 | 1.00 | 7195 |
| *Dist-1* | Trayed Column | 0.35×7.5×10 | $m^3$ | 2.2 | 2729 | 1.00 | 2.00 | 31182 |
| *Dist-2* | Trayed Column | 0.82×21.8×33 | $m^3$ | 1.1 | 12424 | 1.34 | 2.00 | 173670 |
| *HE-3 Economizer* | Heat Exchanger | 6.75 | $m^2$ | 1.013 | 15339 | 1.09 | 1.00 | 48580 |
| *HE-2 Evaporator* | Heat Exchanger | 23.54 | $m^2$ | 1.013 | 16464 | 1.09 | 1.00 | 52144 |
| *HE-1 Super Heater* | Heat Exchanger | 21.43 | $m^2$ | 1.013 | 16,251 | 1.09 | 1.00 | 51469 |
| *HE-6* | Heat Exchanger | 167.1 | $m^2$ | 0.07 | 28747 | 1.09 | 1.00 | 91117 |
| *HE-4* | Heat Exchanger | 7.69 | $m^2$ | 1.013 | 15294 | 1.09 | 1.00 | 48437 |
| *HE-5* | Heat Exchanger | 29.2 | $m^2$ | 1.013 | 17044 | 1.09 | 1.00 | 53980 |
| *HE-7* | Heat Exchanger | 8.62 | $m^2$ | 1.013 | 15284 | 1.09 | 1.00 | 48407 |
| *HE-8* | Heat Exchanger | 58.9 | $m^2$ | 1.013 | 19968 | 1.09 | 1.00 | 63242 |
| *HE-9* | Heat Exchanger | 224.8 | $m^2$ | 75 | 32799 | 1.30 | 1.00 | 114485 |
| *HE-10* | Heat Exchanger | 256.6 | $m^2$ | 75 | 34926 | 1.30 | 1.00 | 121911 |
| *HE-11* | Heat Exchanger | 1.02 | $m^2$ | 4.76 | 20995 | 1.00 | 1.00 | 63546 |
| *HE-Dist1-Con* | Heat Exchanger | 26.5 | $m^2$ | 2.2 | 16767 | 1.02 | 1.00 | 51334 |
| *HE-Dist1-Reb* | Heat Exchanger | 5.67 | $m^2$ | 4.76 | 15458 | 1.00 | 1.00 | 46789 |
| *HE-Dist2-Con* | Heat Exchanger | 49.2 | $m^2$ | 1.1 | 19046 | 1.08 | 1.00 | 60038 |
| *HE-Dist2-Reb* | Heat Exchanger | 32.1 | $m^2$ | 4.76 | 17341 | 1.00 | 1.00 | 52488 |
| | | | | | | | *Total ISBL* | 12630926 |

*Note: Base costs ($C_{p0}$) are in $^{2001} basis from Turton et al.[5] correlations. Bare module costs ($C_{BM}$) converted to €^{2025} using CEPCI = 816/397 and exchange rate 0.92 EUR/USD. $F_p$ = pressure factor; $F_m$ = material factor. Novel equipment (SOEC stack, oxy-combustion boiler) costs from literature sources as previously detailed.*

***Table S24.*** *Complete equipment list and cost breakdown for AWE route.*

| ***Equipment*** | ***Type*** | ***Size*** | ***Unit*** | ***Pressure [bar]*** | ***Base Cost $C_{p0}$ [$]*** | ***$F_p$*** | ***$F_m$*** | ***Bare Module Cost $C_{BM}$ [€]*** |
|---|---|---|---|---|---|---|---|---|
| *AWE Stack* | AWE Cell | 22530 | kW | 10 | 7919295 | 1.00 | 1.00 | 7285751 |
| *Combustion Chamber* | Fired Heater | 8750 | kW | 30 | 2275648 | 1.00 | 1.00 | 2093596 |
| *Reactor* | Fixed Bed Reactor | 78 | $m^3$ | 75 | 53765 | 1.00 | 4.00 | 291341 |
| *PSA* | Adsorption Unit | 5.5 | $m^3$ | 75 | 11306 | 1.00 | 1.00 | 42333 |
| *Dist-1* | Distillation Column | 0.31×10.3 | $m^3$ | 10 | 28160 | 1.00 | 2.00 | 32177 |
| *Dist-2* | Distillation Column | 0.89×24.2 | $m^3$ | 1 | 64900 | 1.41 | 2.00 | 146280 |
| *Flash-1* | Vertical Flash Drum | 0.176 | $m^3$ | 10.5 | 1660 | 1.00 | 1.00 | 6216 |
| *Flash-2* | Vertical Flash Drum | 0.461 | $m^3$ | 75 | 2284 | 1.00 | 1.00 | 8552 |
| *HE-Dist1-Reb* | MPS Reboiler | 7.4 | $m^2$ | 30 | 15303 | 1.12 | 1.00 | 55961 |
| *HE-Dist1-Con* | Total Condenser | 34 | $m^2$ | 1 | 17535 | 1.09 | 1.00 | 52730 |
| *HE-Dist2-Reb* | LPS Reboiler | 21.31 | $m^2$ | 1 | 16330 | 1.05 | 1.00 | 53380 |
| *HE-Dist2-Con* | Total Condenser | 66.91 | $m^2$ | 1 | 18457 | 1.09 | 1.00 | 60328 |
| *HE-1* | Heat Exchanger | 3.1 | $m^2$ | 10 | 16416 | 1.02 | 1.00 | 50149 |
| *HE-2* | Heat Exchanger | 130.7 | $m^2$ | 10 | 26018 | 1.02 | 1.00 | 79483 |
| *HE-3* | $CO_2$/$H_2O$ Cooler | 148.9 | $m^2$ | 10 | 27403 | 1.02 | 1.00 | 83714 |
| *HE-4* | Pure Condenser | 28.8 | $m^2$ | 10 | 17003 | 1.02 | 1.00 | 49262 |
| *HE-5* | Steam/Water Heater | 0.593 | $m^2$ | 1 | 25228 | 1.09 | 1.00 | 79961 |
| *HE-6* | HP Steam Heater | 92.1 | $m^2$ | 40 | 22908 | 1.17 | 1.00 | 75290 |
| *HE-7* | HP Gas/Water Cooler | 266.2 | $m^2$ | 40 | 35557 | 1.17 | 1.00 | 116864 |
| *Gas Turbine* | Turbine | 2016 | kW | 30 | 333868 | 1.00 | 1.00 | 307158 |
| *HP Turbine* | Steam Turbine | 1055 | kW | 120 | 272085 | 1.00 | 2.50 | 250318 |
| *LP Turbine* | Steam Turbine | 2163 | kW | 0.05 | 340048 | 1.00 | 1.00 | 312844 |
| *Compr. CHP* | Compressor | 919 | kW | 30 | 258306 | 1.00 | 1.00 | 237641 |
| *Reactor recycle compressor* | Compressor | 9.8 | kW | 75 | 40400 | 1.00 | 1.00 | 40400 |
| *Process Pump* | Pump | 83 | kW | 10 | 11442 | 1.00 | 1.00 | 34105 |
| *Water Pump* | Pump | 4 | kW | 10 | 3001 | 1.00 | 1.00 | 8944 |
| | | | | | | | *Total ISBL* | 11814381 |

*Note: Base costs ($C_{p0}$) are in $$^{2001}$ basis from Turton et al.[5] correlations. Bare module costs ($C_{BM}$) converted to €$^{2025}$ using CEPCI = 816/397 and exchange rate 0.92 EUR/USD. $F_p$ = pressure factor; $F_m$ = material factor. Novel equipment (AWE unit, combustion chamber) costs from literature sources as previously detailed.*

# S3. Economic Assessment Methodology

This section describes the discounted cash flow (DCF) analysis used to evaluate the economic viability of both process routes, including the calculation of Net Present Value (NPV) and breakeven methanol prices.

## S3.1 Net Present Value (NPV) Calculation

The economic performance of each route was evaluated using a discounted cash flow analysis over the project lifetime. The NPV represents the present value of all future cash flows, discounted at the weighted average cost of capital.

### S3.1.1 Project Timeline and Depreciation

***Table S25.*** *Economic analysis parameters.*

| ***Parameter*** | ***Value*** | ***Unit*** | ***Notes*** |
|---|---|---|---|
| *Total project lifetime* | 15 | years | Including construction and operations |
| *Construction period* | 2 | years | Years 1-2: Capital expenditure phase |
| *Operating period* | 13 | years | Years 3-15: Production phase |
| *Depreciation period* | 10 | years | Linear depreciation over first 10 operating years |
| *Discount rate* | 10 | % | Weighted average cost of capital |
| *Corporate tax rate* | 48 | % | Italian corporate tax rate |
| *Annual operating hours* | 8000 | h/year | Plant availability factor |

*Note: The 2-year construction period (years 1-2) assumes capital is deployed evenly (50% in year 1, 50% in year 2). The 13-year operating period runs from year 3 to year 15.*

### S3.1.2 Annual Cash Flow Calculation

For each operating year $y$ (where $y = 1,2,\ldots,13$ corresponding to project years 3-15), the annual cash flow is calculated according to the procedure described in the following.

Step 1: Annual Revenue

$$Revenue_y = P_{MeOH} \cdot \dot{m}_{MeOH} \quad \text{(S27)}$$

where:

- $P_{MeOH}$ = methanol selling price [€/t];
- $\dot{m}_{MeOH}$ = annual methanol production [t/year].

Step 2: Operating Expenditure (OPEX)

$$OPEX_y = C_{electricity} + C_{biogas} + C_{supplies} + C_{labor} + C_{MTI} + C_{overhead} \quad \text{(S28)}$$

Step 3: Depreciation

Linear depreciation over 10 years:

$$D_y = \begin{cases} FCI/10 & \text{if } y \leq 10 \\ 0 & \text{if } y > 10 \end{cases} \quad \text{(S29)}$$

where $FCI$ is the Fixed Capital Investment.

Step 4: Earnings Before Interest and Tax (*EBIT*)

$$EBIT_y = Revenue_y - OPEX_y - D_y \tag{S30}$$

Step 5: Tax

$$Tax_y = \max(0, \tau \cdot EBIT_y) \tag{S31}$$

where $\tau = 0.48$ is the corporate tax rate. Losses are not taxed.

Step 6: Net Operating Cash Flow

$$CF_{operating,y} = EBIT_y - Tax_y + D_y = Revenue_y - OPEX_y - Tax_y \tag{S32}$$

Step 7: Capital Cash Flows

$$CF_{capital} = \begin{cases} -\dfrac{FCI}{2} & \text{year 1 (construction)} \\ -\dfrac{FCI}{2} - Start\text{-}up - WC & \text{year 2 (construction)} \\ 0 & \text{years 3-14 (operations)} \\ +WC & \text{year 15 (project end, working capital recovery)} \end{cases} \tag{S33}$$

### S3.1.3 NPV calculation

The Net Present Value is calculated by discounting all cash flows to year 0:

$$NPV = \sum_{t=1}^{15} \frac{CF_t}{(1+r)^t} \tag{S34}$$

where:

- $CF_t$ = cash flow in year $t$;
- $r = 0.10$ = discount rate;
- Working Capital (*WC*) is recovered in year 15.

## S3.2 Breakeven Price Calculation

The breakeven methanol price ($P_{BE}$) is defined as the selling price that yields $NPV = 0$ under the baseline assumptions. This price represents the minimum selling price required to achieve the target return on investment (10% discount rate) while recovering all capital and operating costs over the project lifetime.

### S3.2.1 Breakeven Price Decomposition

To facilitate understanding of the economic drivers, the breakeven price can be decomposed into operating and capital components:

$$P_{BE} = \frac{OPEX_{annual}}{\dot{m}_{MeOH}} + \frac{Capital\ Component}{\dot{m}_{MeOH}} \quad \text{(S35)}$$

where the Capital Component represents the levelized cost contribution required to recover the initial capital investment (CAPEX), provide the target 10% return on investment, account for tax shields from depreciation, and account for working capital timing effects.

***Table S26.*** *Breakeven price decomposition for Italian baseline scenario (177.4 €/MWh electricity, 60 €/MWh biogas, 884 €/t methanol benchmark).*

| *Component* | *AWE Route* | *SOEC Route* | *Unit* |
|---|---|---|---|
| *Operating Costs* | | | |
| *Annual OPEX* | 33.60 | 27.86 | M€/year |
| *Annual production* | 19689 | 17512 | t/year |
| *OPEX per ton* | 1707 | 1592 | €/t |
| *Capital Contribution* | | | |
| *Total CAPEX* | 25.84 | 27.63 | M€ |
| *Breakeven Price (NPV = 0)* | 2020 | 1919 | €/t |

*Note: The capital component per ton is derived as the residual when NPV = 0 is achieved. It represents the levelized capital contribution that includes capital recovery at 10% return, plus all tax effects, depreciation shields, and working capital timing from the full DCF model. The SOEC route has a higher capital component per ton due to lower production volume, despite similar total CAPEX. However, SOEC route significantly lower OPEX per ton results in a slightly lower overall breakeven price.*

## S3.2.2 Sensitivity Analysis

Sensitivity analyses were performed by varying individual parameters while holding others constant at baseline values. The parameters investigated include:

- methanol price: 600 to 2600 €/t;
- electricity price: 40 to 300 €/$MWh_{el}$;
- biogas price: 30 to 120 €/$MWh_{th}$;
- CAPEX multiplier: from 0.4× to 2.5× of the baseline values;
- grid carbon intensity: from 7 to 585 g $CO_{2e}$/$kWh_{el}$ (European range of values).

For each sensitivity case, NPV was recalculated by re-evaluating the full economic model with the modified parameter. Breakeven prices were determined by solving the NPV = 0 equivalence for each scenario.

## S3.3 Geographic Analysis Across EU Countries

The economic and environmental performance of both routes was evaluated across all 27 European member states using country-specific data for electricity price [€/$MWh_{el}$] (Source: Eurostat[6], industrial tariffs), and electric grid carbon intensity [g $CO_{2e}$/$kWh_{el}$] (Source: EEA[7]). All other parameters (biogas price, CAPEX, labor costs, methanol price) were held constant at Italian baseline values to isolate the effects of electricity price and carbon intensity.

***Table S27.*** *EU country input data for geographic analysis (all 27 European member states, sorted by grid carbon intensity).*

| ***Country*** | ***Grid CI [g $CO_{2e}$/$kWh_{el}$]*** | ***Electricity Price [€/$MWh_{el}$]*** |
|---|---:|---:|
| *Sweden* | 7 | 95.8 |
| *Lithuania* | 29 | 159.8 |
| *Finland* | 37 | 79.7 |
| *Luxembourg* | 37 | 176.3 |
| *France* | 43 | 137.2 |
| *Austria* | 55 | 178.2 |
| *Portugal* | 72 | 113.6 |
| *Slovakia* | 75 | 163.8 |
| *Denmark* | 76 | 122.6 |
| *Latvia* | 82 | 135.4 |
| *Spain* | 129 | 124.0 |
| *Hungary* | 137 | 192.8 |
| *Croatia* | 141 | 216.6 |
| *Belgium* | 145 | 159.0 |
| *Slovenia* | 172 | 141.0 |
| ***Italy*** | **180** | **177.4** |
| *Netherlands* | 235 | 163.3 |
| *Ireland* | 238 | 263.6 |
| *Greece* | 285 | 168.5 |
| *Romania* | 296 | 156.0 |
| *Germany* | 298 | 182.8 |
| *Bulgaria* | 301 | 145.3 |
| *Czechia* | 332 | 164.7 |
| *Malta* | 337 | 133.3 |
| *Cyprus* | 535 | 164.3 |
| *Poland* | 554 | 129.5 |
| *Estonia* | 585 | 133.5 |

***Table S28.*** *Economic performance results by country (baseline methanol price 884 €/t).*

| ***Country*** | ***Grid CI [g $CO_{2e}$/kWh$_{el}$]*** | ***Elec Price [€/MWh$_{el}$]*** | ***NPV AWE [M€]*** | ***NPV SOEC [M€]*** | ***BE AWE [€/t]*** | ***BE SOEC [€/t]*** |
|---|---|---|---|---|---|---|
| *Sweden* | 7 | 95.8 | -53.67 | -45.33 | 1394 | 1390 |
| *Lithuania* | 29 | 159.8 | -116.16 | -92.28 | 1885 | 1805 |
| *Finland* | 37 | 79.7 | -37.95 | -33.52 | 1270 | 1286 |
| *Luxembourg* | 37 | 176.3 | -132.27 | -104.38 | 2012 | 1912 |
| *France* | 43 | 137.2 | -94.09 | -75.70 | 1712 | 1659 |
| *Austria* | 55 | 178.2 | -134.13 | -105.77 | 2026 | 1925 |
| *Portugal* | 72 | 113.6 | -71.05 | -58.38 | 1530 | 1506 |
| *Slovakia* | 75 | 163.8 | -120.07 | -95.21 | 1916 | 1831 |
| *Denmark* | 76 | 122.6 | -79.84 | -64.99 | 1599 | 1564 |
| *Latvia* | 82 | 135.4 | -92.34 | -74.38 | 1698 | 1647 |
| *Spain* | 129 | 124.0 | -81.21 | -66.01 | 1610 | 1573 |
| *Hungary* | 137 | 192.8 | -148.38 | -116.48 | 2139 | 2019 |
| *Croatia* | 141 | 216.6 | -171.62 | -133.94 | 2321 | 2174 |
| *Belgium* | 145 | 159.0 | -115.38 | -91.69 | 1879 | 1800 |
| *Slovenia* | 172 | 141.0 | -97.80 | -78.48 | 1741 | 1683 |
| ***Italy*** | **180** | **177.4** | **-133.35** | **-105.19** | **2020** | **1919** |
| *Netherlands* | 235 | 163.3 | -119.58 | -94.84 | 1912 | 1828 |
| *Ireland* | 238 | 263.6 | -217.51 | -168.42 | 2682 | 2479 |
| *Greece* | 285 | 168.5 | -124.66 | -98.66 | 1952 | 1862 |
| *Romania* | 296 | 156.0 | -112.45 | -89.49 | 1856 | 1781 |
| *Germany* | 298 | 182.8 | -138.62 | -109.15 | 2062 | 1955 |
| *Bulgaria* | 301 | 145.3 | -102.00 | -81.64 | 1774 | 1711 |
| *Czechia* | 332 | 164.7 | -120.94 | -95.87 | 1923 | 1837 |
| *Malta* | 337 | 133.3 | -90.29 | -72.84 | 1682 | 1633 |
| *Cyprus* | 535 | 164.3 | -120.55 | -95.58 | 1920 | 1834 |
| *Poland* | 554 | 129.5 | -86.58 | -70.05 | 1652 | 1609 |
| *Estonia* | 585 | 133.5 | -90.48 | -72.98 | 1683 | 1635 |

## S3.4 Sensitivity Analysis Results

This section provides detailed sensitivity analysis results for CAPEX, biogas price, and methanol price variations.

***Table S29.*** *CAPEX sensitivity analysis - NPV vs electrolyzer specific cost (Italian baseline: 177.4 €/MWh$_{el}$ electricity, 60 €/MWh$_{el}$ biogas, 884 €/t methanol).*

| *AWE CAPEX [€/kW]* | *NPV AWE [M€]* | *SOEC CAPEX [€/kW]* | *NPV SOEC [M€]* |
|---|---|---|---|
| *128* | -119.38 | 208 | -90.07 |
| *160* | -121.71 | 260 | -92.59 |
| *192* | -124.04 | 312 | -95.11 |
| *224* | -126.36 | 364 | -97.63 |
| *256* | -128.69 | 416 | -100.15 |
| *288* | -131.02 | 468 | -102.67 |
| ***320*** | **-133.35** | **520** | **-105.19** |
| *352* | -135.67 | 572 | -107.71 |
| *384* | -138.00 | 624 | -110.22 |
| *416* | -140.33 | 676 | -112.74 |
| *448* | -142.65 | 728 | -115.26 |
| *480* | -144.98 | 780 | -117.78 |
| *512* | -147.31 | 832 | -120.30 |
| *544* | -149.63 | 884 | -122.82 |
| *576* | -151.96 | 936 | -125.34 |
| *608* | -154.29 | 988 | -127.86 |
| *640* | -156.61 | 1040 | -130.37 |
| *672* | -158.94 | 1092 | -132.89 |
| *704* | -161.27 | 1144 | -135.41 |
| *736* | -163.59 | 1196 | -137.93 |
| *768* | -165.92 | 1248 | -140.45 |
| *800* | -168.25 | 1300 | -142.97 |

*Note: Baseline values are 320 €/kW for AWE and 520 €/kW for SOEC (shown in **bold**).*

***Table S30.*** *Biogas price sensitivity analysis (Italian baseline: 177.4 €/MWh electricity, 884 €/t methanol).*

| *Biogas Price (€/MWh)* | *NPV AWE (M€)* | *NPV SOEC (M€)* |
|---|---|---|
| *30* | -118.28 | -90.12 |
| *35* | -120.79 | -92.63 |
| *40* | -123.30 | -95.14 |
| *45* | -125.81 | -97.65 |
| *50* | -128.32 | -100.16 |
| *55* | -130.83 | -102.68 |
| ***60*** | **-133.35** | **-105.19** |
| *65* | -135.86 | -107.70 |
| *70* | -138.37 | -110.21 |
| *75* | -140.88 | -112.72 |
| *80* | -143.39 | -115.23 |
| *85* | -145.90 | -117.74 |
| *90* | -148.41 | -120.25 |
| *95* | -150.92 | -122.77 |
| *100* | -153.44 | -125.28 |
| *105* | -155.95 | -127.79 |
| *110* | -158.46 | -130.30 |
| *115* | -160.97 | -132.81 |
| *120* | -163.48 | -135.32 |

*Note: Baseline biogas price is 60 €/MWh (shown in **bold**).*

***Table S31.*** *Methanol price sensitivity analysis across EU countries - NPV at four price points.*

| ***Country*** | ***NPV AWE (M€)*** | ***NPV SOEC (M€)*** | ***NPV AWE (M€)*** | ***NPV SOEC (M€)*** | ***NPV AWE (M€)*** | ***NPV SOEC (M€)*** | ***NPV AWE (M€)*** | ***NPV SOEC (M€)*** |
|---|---|---|---|---|---|---|---|---|
| ***Methanol price*** | ***884 €/t*** | | ***1200 €/t*** | | ***1700 €/t*** | | ***2500 €/t*** | |
| *Sweden* | -53.67 | -45.33 | -14.15 | -11.18 | 20.26 | 18.22 | 73.15 | 65.26 |
| *Lithuania* | -116.16 | -92.28 | -75.98 | -56.54 | -13.14 | -6.19 | 40.65 | 40.85 |
| *Finland* | -37.95 | -33.52 | -4.63 | -5.04 | 28.43 | 24.36 | 81.32 | 71.41 |
| *Luxembourg* | -132.27 | -104.38 | -92.09 | -68.64 | -28.52 | -12.95 | 32.28 | 34.56 |
| *France* | -94.09 | -75.70 | -53.92 | -39.96 | -0.76 | 2.43 | 52.13 | 49.47 |
| *Austria* | -134.13 | -105.77 | -93.95 | -70.04 | -30.38 | -14.26 | 31.31 | 33.83 |
| *Portugal* | -71.05 | -58.38 | -30.87 | -22.82 | 11.22 | 11.43 | 64.11 | 58.47 |
| *Slovakia* | -120.07 | -95.21 | -79.89 | -59.47 | -16.79 | -7.72 | 38.62 | 39.33 |
| *Denmark* | -79.84 | -64.99 | -39.66 | -29.25 | 6.65 | 8.00 | 59.54 | 55.04 |
| *Latvia* | -92.34 | -74.38 | -52.16 | -38.64 | 0.15 | 3.12 | 53.04 | 50.16 |
| *Spain* | -81.21 | -66.01 | -41.03 | -30.28 | 5.94 | 7.46 | 58.83 | 54.51 |
| *Hungary* | -148.38 | -116.48 | -108.20 | -80.75 | -44.63 | -24.27 | 23.90 | 28.26 |
| *Croatia* | -171.62 | -133.94 | -131.44 | -98.21 | -67.87 | -41.67 | 11.81 | 19.18 |
| *Belgium* | -115.38 | -91.69 | -75.20 | -55.95 | -12.41 | -5.89 | 41.06 | 41.16 |
| *Slovenia* | -97.80 | -78.48 | -57.63 | -42.75 | -2.69 | 0.98 | 50.20 | 48.02 |
| ***Italy*** | **-133.35** | **-105.19** | **-93.17** | **-69.45** | **-29.60** | **-13.71** | **31.72** | **34.14** |
| *Netherlands* | -119.58 | -94.84 | -79.40 | -59.11 | -16.33 | -7.53 | 38.88 | 39.52 |
| *Ireland* | -217.51 | -168.42 | -177.33 | -132.69 | -113.76 | -76.14 | -12.79 | 1.26 |
| *Greece* | -124.66 | -98.66 | -84.48 | -62.92 | -21.08 | -9.51 | 36.24 | 37.53 |
| *Romania* | -112.45 | -89.49 | -72.27 | -53.75 | -10.31 | -4.74 | 42.58 | 42.30 |
| *Germany* | -138.62 | -109.15 | -98.44 | -73.41 | -34.87 | -17.41 | 28.98 | 32.08 |
| *Bulgaria* | -102.00 | -81.64 | -61.83 | -45.90 | -4.88 | -0.66 | 48.01 | 46.38 |
| *Czechia* | -120.94 | -95.87 | -80.77 | -60.14 | -17.61 | -8.06 | 38.16 | 38.98 |
| *Malta* | -90.29 | -72.84 | -50.11 | -37.10 | 1.22 | 3.92 | 54.11 | 50.96 |
| *Cyprus* | -120.55 | -95.58 | -80.38 | -59.84 | -17.25 | -7.91 | 38.37 | 39.13 |
| *Poland* | -86.58 | -70.05 | -46.40 | -34.31 | 3.14 | 5.37 | 56.04 | 52.41 |
| *Estonia* | -90.48 | -72.98 | -50.30 | -37.25 | 1.11 | 3.84 | 54.01 | 50.88 |

# S4. Carbon Accounting Methodology

This section describes the gate-to-gate carbon accounting framework used to evaluate the environmental performance of both process routes, including the calculation of carbon intensity and the treatment of biogenic carbon flows.

## S4.1 Carbon Intensity Calculation Framework

The carbon intensity (*CI*) of methanol production was calculated using a gate-to-gate system boundary following the methodology described in Scomazzon et al.[8] for bio-based e-fuels. *CI* quantifies the net greenhouse gas emissions per unit of methanol product and is expressed as t $CO_{2e}$ per ton of methanol. The equation for calculating *CI* is:

$$CI = \frac{E_1 + E_2 + E_3 - E_4}{Production_{MeOH}} \quad \text{(S36)}$$

where:

- $E_1$: Emissions from biogas feedstock production (upstream, outside gate) [t $CO_{2e}$ /year];

- $E_2$: Direct $CO_2$ emissions from combustion processes (burner, flare) [t $CO_{2e}$ /year];
- $E_3$: Indirect emissions from grid electricity consumption [t $CO_{2e}$ /year];
- $E_4$: Carbon credit for biogenic carbon sequestered in methanol product [t $CO_{2e}$ /year];
- $Production_{MeOH}$: Annual methanol production [t/year].

Emissions from biogas feedstock production were calculated assuming a biogas carbon intensity of 18 $gCO_{2e}$/MJ of biogas, according to:

$$E_1 = 18 \cdot \dot{m}_{\text{biogas}} \cdot \text{HHV}_{\text{biogas}} \cdot h_{\text{operating}} \tag{S37}$$

where $\dot{m}_{\text{biogas}}$ is the inlet biogas mass flow rate [kg/h], $\text{HHV}_{\text{biogas}}$ is the biogas higher heating value (19.6 [MJ/kg]), and $h_{operating}$ = 8000 h/year.

Direct emissions arise from combustion of tail gases (PSA off-gas, light ends from distillation) in the process burner:

$$E_2 = \dot{m}_{\text{CO}_2,\text{direct}} \cdot h_{\text{operating}} \tag{S38}$$

where $\dot{m}_{\text{CO}_2,\text{direct}}$is the mass flow rate of $CO_2$ emitted from the burner [t/h].

Indirect emissions derive from net grid electricity consumption and are calculated as:

$$E_3 = P_{\text{net}} \cdot h_{\text{operating}} \cdot CI_{grid} \tag{S39}$$

where:

- $P_{net}$ = Net electrical power imported from grid [$MW_{el}$];
- $CI_{grid}$ = Grid carbon intensity [t of $CO_{2e}$ /$MWh_{el}$];
- $h_{operating}$ = 8000 h/year.

Finally, the biogenic carbon credit depends on the end-use application of methanol:

- Chemical use: methanol used as chemical feedstock (e.g., formaldehyde, acetic acid production) sequesters biogenic carbon for extended periods. The carbon credit equals the total biogenic carbon captured in the product, where the factor 44/32 converts methanol mass to carbon dioxide content (1 mol $CO_2$ per mol $CH_3OH$):

  $$E_4^{\text{chem}} = \dot{m}_{\text{MeOH}} \cdot \frac{44}{32} \cdot h_{\text{operating}} \tag{S40}$$

- Fuel use: methanol used as fuel (e.g., maritime, transportation) releases all carbon back to atmosphere upon combustion. No long-term sequestration credit applies:

  $$E_4^{\text{fuel}} = 0 \tag{S41}$$

## S4.2 Carbon Intensity Results

***Table S32.*** *Carbon intensity for the 27 European countries (methanol deployed as chemical feedstock with biogenic credit).*

| ***Country*** | ***Grid CI [g of $CO_{2e}$/kWh$_{el}$]*** | ***CI AWE (methanol chem) [t of $CO_{2e}$/t]*** | ***CI SOEC (methanol chem) [t of $CO_{2e}$/t]*** |
|---|---|---|---|
| *Sweden* | 7 | -1.004 | -0.973 |
| *Lithuania* | 29 | -0.835 | -0.830 |
| *Finland* | 37 | -0.774 | -0.778 |
| *Luxembourg* | 37 | -0.774 | -0.778 |
| *France* | 43 | -0.727 | -0.739 |
| *Austria* | 55 | -0.635 | -0.662 |
| *Portugal* | 72 | -0.505 | -0.551 |
| *Slovakia* | 75 | -0.482 | -0.532 |
| *Denmark* | 76 | -0.474 | -0.525 |
| *Latvia* | 82 | -0.428 | -0.486 |
| *Spain* | 129 | -0.067 | -0.182 |
| *Hungary* | 137 | -0.006 | -0.130 |
| *Croatia* | 141 | 0.025 | -0.104 |
| *Belgium* | 145 | 0.056 | -0.078 |
| *Slovenia* | 172 | 0.263 | 0.097 |
| ***Italy*** | **180** | **0.325** | **0.149** |
| *Netherlands* | 235 | 0.747 | 0.506 |
| *Ireland* | 238 | 0.770 | 0.526 |
| *Greece* | 285 | 1.131 | 0.830 |
| *Romania* | 296 | 1.215 | 0.902 |
| *Germany* | 298 | 1.231 | 0.915 |
| *Bulgaria* | 301 | 1.254 | 0.934 |
| *Czechia* | 332 | 1.492 | 1.135 |
| *Malta* | 337 | 1.530 | 1.168 |
| *Cyprus* | 535 | 3.051 | 2.452 |
| *Poland* | 554 | 3.197 | 2.575 |
| *Estonia* | 585 | 3.435 | 2.777 |

*Note: Negative values indicate net carbon sequestration (carbon-negative methanol).*

***Table S33.*** *Carbon intensity for the 27 European countries (methanol deployed as fuel).*

| ***Country*** | ***Grid CI [g of $CO_{2e}$/$kWh_{el}$]*** | ***CI AWE (methanol fuel) [t of $CO_{2e}$/t]*** | ***CI SOEC (methanol fuel) [t of $CO_{2e}$/t]*** |
|---|---:|---:|---:|
| *Sweden* | 7 | 0.370 | 0.401 |
| *Lithuania* | 29 | 0.539 | 0.543 |
| *Finland* | 37 | 0.600 | 0.595 |
| *Luxembourg* | 37 | 0.600 | 0.595 |
| *France* | 43 | 0.646 | 0.634 |
| *Austria* | 55 | 0.738 | 0.712 |
| *Portugal* | 72 | 0.869 | 0.822 |
| *Slovakia* | 75 | 0.892 | 0.842 |
| *Denmark* | 76 | 0.900 | 0.848 |
| *Latvia* | 82 | 0.946 | 0.887 |
| *Spain* | 129 | 1.307 | 1.192 |
| *Hungary* | 137 | 1.368 | 1.244 |
| *Croatia* | 141 | 1.399 | 1.270 |
| *Belgium* | 145 | 1.429 | 1.296 |
| *Slovenia* | 172 | 1.637 | 1.471 |
| ***Italy*** | **180** | **1.698** | **1.523** |
| *Netherlands* | 235 | 2.121 | 1.880 |
| *Ireland* | 238 | 2.144 | 1.899 |
| *Greece* | 285 | 2.505 | 2.204 |
| *Romania* | 296 | 2.589 | 2.275 |
| *Germany* | 298 | 2.604 | 2.288 |
| *Bulgaria* | 301 | 2.627 | 2.308 |
| *Czechia* | 332 | 2.866 | 2.509 |
| *Malta* | 337 | 2.904 | 2.541 |
| *Cyprus* | 535 | 4.424 | 3.826 |
| *Poland* | 554 | 4.570 | 3.949 |
| *Estonia* | 585 | 4.808 | 4.150 |

*Note: For fuel applications (no biogenic credit), both routes show substantial emissions, exceeding fossil methanol benchmarks (0.54-0.76 t* $CO_{2e}$*/t) in countries with grid carbon intensity above ~200 g/*$kWh_{el}$*.*

# S5. Stream properties and composition

**Table S34**. SOEC route properties and composition. Temperature (*T* [°C]), pressure (*P* [bar]), mass flow rate (*m* [kg/h]), molar flow rate (*n* [kmol/h]), molar fractions of species *i* ($x_i$ [-]) of the main process streams. Stream numbers referred to Figure 2 of the main text.

| | | | | | $x_i$ | | | | | | | |
|---|---|---|---|---|---|---|---|---|---|---|---|---|
| | ***T*** | ***P*** | ***m*** | ***n*** | **$CO_2$** | **CO** | **$H_2$** | **$H_2O$** | **$CH_4$** | **Methanol** | **$O_2$** | **Others** |
| Unit | [°C] | [bar] | [kg/h] | [kmol/h] | [-] | [-] | [-] | [-] | [-] | [-] | [-] | [-] |
| S-1 | 20.0 | 1.0 | 2271.6 | 83.4 | 0.40 | 0.00 | 0.00 | 0.00 | 0.60 | 0.00 | 0.00 | 0.00 |
| S-2 | 1700.1 | 1.0 | 14056.0 | 533.8 | 0.32 | 0.00 | 0.00 | 0.68 | 0.00 | 0.00 | 0.00 | 0.00 |
| S-5 | 800.0 | 1.1 | 14056.0 | 533.8 | 0.32 | 0.00 | 0.00 | 0.68 | 0.00 | 0.00 | 0.00 | 0.00 |
| S-10 | 120.0 | 1.0 | 8581.1 | 350.3 | 0.25 | 0.00 | 0.00 | 0.75 | 0.00 | 0.00 | 0.00 | 0.00 |
| S-18 | 800.0 | 1.1 | 7603.9 | 316.9 | 0.27 | 0.05 | 0.10 | 0.58 | 0.00 | 0.00 | 0.00 | 0.00 |
| S-19 | 20.0 | 1.0 | 3203.3 | 100.1 | 0.00 | 0.00 | 0.00 | 0.00 | 0.00 | 0.00 | 1.00 | 0.00 |
| S-20 | 800.0 | 1.1 | 4143.0 | 315.4 | 0.06 | 0.26 | 0.56 | 0.12 | 0.00 | 0.00 | 0.00 | 0.00 |
| S-30 | 220.0 | 75.0 | 12896.1 | 1148.2 | 0.08 | 0.21 | 0.69 | 0.00 | 0.01 | 0.00 | 0.00 | 0.00 |
| S-31 | 250.0 | 74.6 | 12896.1 | 1007.6 | 0.09 | 0.18 | 0.64 | 0.01 | 0.01 | 0.07 | 0.00 | 0.00 |
| S-45 | 65.0 | 1.1 | 2181.0 | 68.0 | 0.00 | 0.00 | 0.00 | 0.00 | 0.00 | 1.00 | 0.00 | 0.00 |

**Table S35**. AWE route properties and composition. Temperature (*T* [°C]), pressure (*P* [bar]), mass flow rate (*m* [kg/h]), molar flow rate (*n* [kmol/h]), molar ratios of species *i* ($x_i$ [-]) of the main process streams. Stream numbers referred to Figure 3 of the main text.

| | | | | | $x_i$ | | | | | | | |
|---|---|---|---|---|---|---|---|---|---|---|---|---|
| | ***T*** | ***P*** | ***m*** | ***n*** | **$CO_2$** | **CO** | **$H_2$** | **$H_2O$** | **$CH_4$** | **Methanol** | **$O_2$** | **Others** |
| Unit | [°C] | [bar] | [kg/h] | [kmol/h] | [-] | [-] | [-] | [-] | [-] | [-] | [-] | [-] |
| S-1 | 20.0 | 1.0 | 2271.6 | 83.4 | 0.40 | 0.00 | 0.00 | 0.00 | 0.60 | 0.00 | 0.00 | 0.00 |
| S-2 | 170.4 | 30.0 | 27166.4 | 677.9 | 0.78 | 0.00 | 0.00 | 0.01 | 0.07 | 0.00 | 0.14 | 0.00 |
| S-3 | 1278.1 | 30.0 | 27166.4 | 677.9 | 0.85 | 0.00 | 0.00 | 0.15 | 0.00 | 0.00 | 0.00 | 0.00 |
| S-7 | 40.0 | 10.5 | 27166.4 | 677.9 | 0.85 | 0.00 | 0.00 | 0.15 | 0.00 | 0.00 | 0.00 | 0.00 |
| S-10 | 40.0 | 10.5 | 3571.2 | 81.5 | 0.99 | 0.00 | 0.00 | 0.01 | 0.00 | 0.00 | 0.00 | 0.00 |
| S-20 | 60.0 | 10.0 | 4392.9 | 243.8 | 0.00 | 0.00 | 0.00 | 1.00 | 0.00 | 0.00 | 0.00 | 0.00 |
| S-26 | 60.0 | 10.0 | 537.4 | 241.4 | 0.00 | 0.00 | 0.99 | 0.01 | 0.00 | 0.00 | 0.00 | 0.00 |
| S-27 | 52.0 | 10.0 | 4117.4 | 327.2 | 0.25 | 0.00 | 0.74 | 0.01 | 0.00 | 0.00 | 0.00 | 0.00 |
| S-34 | 220.0 | 75.0 | 11802.5 | 1334.6 | 0.13 | 0.01 | 0.83 | 0.00 | 0.00 | 0.00 | 0.02 | 0.01 |
| S-35 | 232.9 | 75.0 | 11802.5 | 1175.9 | 0.08 | 0.01 | 0.74 | 0.07 | 0.00 | 0.07 | 0.02 | 0.01 |
| S-43 | 40.0 | 1.0 | 2461.15 | 76.66 | 0.00 | 0.00 | 0.00 | 0.00 | 0.00 | 0.995 | 0.00 | 0.005 |

## S6. List of symbols

### Acronyms

- AWE Alkaline water electrolysis
- BOP Balance of plant
- CAPEX Capital expenditure
- CEPCI Chemical Engineering Plant Cost Index
- CHP Combined heat and power
- DCF Discounted cash flow
- e-MeOH Electro-methanol
- FCI Fixed capital investment
- HE Heat exchanger
- HP High pressure
- IEA International Energy Agency
- ISBL Inside battery limits
- KOH Potassium hydroxide
- LHV Lower heating value
- LMTD Log-mean temperature difference
- LP Low pressure
- MTI Maintenance, taxes, and insurance
- NETL National Energy Technology Laboratory
- NPV Net present value
- OPEX Operating expenditure
- OSBL Outside battery limits
- PFR Plug flow reactor
- PSA Pressure swing adsorption
- RStoic Stoichiometric reactor (Aspen Plus)
- RU Reactant utilization
- SOEC Solid oxide electrolysis cell
- TDC Total direct cost
- WC Working capital

### Mathematical symbols

- $A$ Heat transfer area [$m^2$]
- $C$ Cost [€ or $]

- $C_{biogas}$ Annual cost of biogas feedstock [€/year]
- $C_{BM}$ Bare module cost [€]
- $C_{electricity}$ Annual cost of electricity [€/year]
- $C_{labor}$ Annual labor cost [€/year]
- $C_{p0}$ Base equipment cost at ambient pressure and carbon steel construction [€]
- $C_{supplies}$ Annual cost of operating supplies [€/year]
- $\Delta H$ Reaction enthalpy
- $\Delta P$ Pressure drop
- $\Delta T_{lm}$ Log-mean temperature difference [K or °C]
- $\Delta T_{min}$ Minimum temperature approach [°C]
- $E_{annual}$ Annual net electricity consumption [MWh/year]
- $\eta$ Efficiency
- $\eta_{carbon}$ Carbon efficiency
- $F_{BM}$ Bare module factor [-]
- $F_m$ Material factor
- $F_p$ Pressure factor
- $f_{supervision}$ Supervision cost factor
- $h_{operating}$ Annual operating hours [h/year]
- $K_i$ Equipment-specific cost constants
- $k_i$ Reaction rate constant
- $N_{OL}$ Number of operators per shift
- $N_{np}$ Number of non-particulate processing steps
- $N_{workers}$ Total number of plant workers
- $P$ Pressure [bar or barg]
- $P_{net}$ Net electrical power demand [MW]
- $p_i$ Partial pressure of component i [bar]
- $Q$ Heat duty [MW]
- $R$ Universal gas constant
- $RR$ Recycle ratio
- $T$ Temperature [K or °C]
- $U$ Overall heat transfer coefficient [W m$^{-2}$ K$^{-1}$]
- $v$ Stoichiometric coefficient
- $x_i$ Mole fraction of species $i$ [0.%]

## S7. Supplementary References

(1) Rajaee, F.; Romano, M.C.; Ritvanen, J., Flexible integrated gasification solid oxide cell (IGSOC) plant for bio-methanol and bio-power generation. Energy 2025, 337, 138457. https://doi.org/10.1016/j.energy.2025.138457.

(2) Yao, J.; Kraussler, M.; Benedikt, F.; Hofbauer, H. Techno-economic assessment of hydrogen production based on dual fluidized bed biomass steam gasification, biogas steam reforming, and alkaline water electrolysis processes. Energy Convers. Manage. 2017, 145, 278−292. https://doi.org/10.1016/j.enconman.2017.04.084.

(3) Zhou, H.; Cao, A.; Meng, W.; Wang, D.; Li, G.; Yang, S. Process integration and analysis of coupling solid oxide electrolysis cell (SOEC) and $CO_2$ to methanol. Energy 2024, 307, 132652. https://doi.org/10.1016/j.energy.2024.132652.

(4) NETL. Cost and performance baseline for fossil energy plants volume 1a: Bituminous coal (PC) and natural gas to electricity revision 3 (Report No. DOE/NETL-2015/1723) https://netl.doe.gov/sites/default/files/2020-02/Modular-Staged-Pressurized-Oxy-combustion-Power-Plant-System-Washington-University-in-St.-Louis.pdf. 2019 (accessed Dec 18, 2025).

(5) Turton, R.; Shaeiwitz, J.A.; Bhattacharyya, D.; Whiting, W.B. Analysis, Synthesis, and Design of Chemical Processes. Pearson Education 2018, Fifth ed.

(6) Eurostat. Electricity price statistics-non-household consumers (band IC, excluding taxes and levies) https://ec.europa.eu/eurostat/statistics-explained/index.php?title=Electricity_price_statistics. 2025 (accessed Dec 18, 2025).

(7) EEA. Greenhouse gas emission intensity of electricity generation, country level https://www.eea.europa.eu/en/analysis/indicators/greenhouse-gas-emission-intensity-of-1. 2024 (accessed Dec 18, 2025).

(8) Scomazzon, M.; Barbera, E.; Bezzo, F. Alternative sustainable routes to methanol production: Techno-economic and environmental assessment. J. Environ. Chem. Eng. 2024, 12, 112674. https://doi.org/10.1016/j.jece.2024.112674.
